# PLPP/CIN-mediated NEDD4-2 S448 dephosphorylation regulates neuronal excitability via GluA1 ubiquitination

**DOI:** 10.1038/s41419-019-1781-0

**Published:** 2019-07-18

**Authors:** Ji-Eun Kim, Duk-Shin Lee, Min Ju Kim, Tae-Cheon Kang

**Affiliations:** Department of Anatomy and Neurobiology, Institute of Epilepsy Research, College of Medicine, Hallym University, Chunchon, 24252 South Korea

**Keywords:** Cell death in the nervous system, Epilepsy

## Abstract

Neuronal precursor cell expressed developmentally downregulated 4-2 (NEDD4-2) is an E3 ubiquitin ligase to regulate ion transport by controlling cellular trafficking/endocytosis and lysosomal degradation of ion channels and transporters. Thus, NEDD4-2 is relevant to neuronal excitability and epileptic encephalopathies in human patients. However, the regulatory molecules for NEDD4-2 dephosphorylation have been still elusive. Here, we demonstrate that pyridoxal-5′-phosphate phosphatase/chronophin (PLPP/CIN) specifically dephosphorylated NEDD4-2 serine (S) 448 site. PLPP/CIN deletion inhibited NEDD4-2 ubiquitination, and diminished the responsiveness of α‐amino‐3‐hydroxy‐5‐methyl‐4‐isoxazolepropionic acid receptor (AMPAR) by facilitating NEDD4-2-mediated ubiquitination of GluA1 subunit under physiological condition. PLPP/CIN overexpression reversed these effects. These PLPP/CIN-mediated processes were required for the increased seizure severity and its progression in response to kainic acid (KA). Therefore, we suggest the novel function of PLPP/CIN as a NEDD4-2 phosphatase, which may be a potential therapeutic target for NEDD4-2-associated diseases as well as various neurological and psychiatric disorders, including epilepsy.

## Introduction

Epilepsy is one of the common neurological disorders, suffering about 1% of all people, which is characterized by the periodic occurrence of seizures exhibiting abnormal synchronized neuronal discharges. Unprovoked recurrent seizures contribute to a cyclical or progressive process of worsening epilepsy and neurological deficits including learning disabilities and memory problems. The pathophysiology underlying seizure susceptibility is relevant to channelopathy, aberrant synaptic organization, impaired glial function, inflammation, and neuronal loss^1–9^. However, the cellular and molecular mechanisms in epilepsy remain unclear.

Ubiquitination is a rapid, local, and reversible posttranslational modification that involves the covalent conjugation of ubiquitin to target proteins, which assigns misfolded cytosolic proteins for degradation by the 26S proteasome and regulates physiological processes. Ubiquitination is involved in modulation of synaptic function via nonproteolytic processes such as endocytosis, protein localization/targeting, complex assembly and regulation of the duration, and intensity of signaling by effector molecules. Generally, ubiquitin is bound to lysine residues on target proteins by a cascade of reactions carried out sequentially by the ubiquitin-activating enzyme (E1), ubiquitin-conjugating enzyme (E2), and ubiquitin ligase enzyme (E3). In the final step, an E3 ubiquitin ligase transfers the ubiquitin to recognition motifs in the target proteins and thus confers target protein specificity^10,11^.

Neuronal precursor cell expressed developmentally downregulated 4-2 (NEDD4-2) is an E3 ubiquitin ligase to control cellular trafficking/endocytosis and lysosomal degradation of ion channels and transporters^12–17^. Therefore, it is required for optimal regulation of NEDD4-2-mediated channel degradation/internalization to maintain neuronal excitability. Indeed, NEDD4-2 mutations are relevant to epileptic encephalopathies in human patients^18–20^. In addition, *Nedd4-2*^*andi*^ mice, in which the long-form (isoform 1) of NEDD4-2 is selectively deleted due to a spontaneous mutation in exon-2, show the elevated kainic acid (KA)-induced seizure susceptibility. The fidelity and activation of NEDD4-2 activity is regulated by phosphorylations that which disrupt target channels-NEDD4-2 interactions^21,22^. Oppositely, NEDD4-2 phosphorylations are required for a substrate ubiquitination^23,24^ and maintenance of its stability^12^. Although the phosphorylation of human NEDD4-2 on serine (S) 342 (equivalent to S338 in *Xenopus* NEDD4-2, S222 in murine NEDD4-2) and S448 (corresponding to S444 in *Xenopus* NEDD4-2, S328 in murine NEDD4-2) residues by various kinases including serum glucocorticoid kinase (SGK1)^25–27^, furthermore, little is known yet to explain the regulatory molecules for NEDD4-2 dephosphorylation.

Pyridoxal-5′-phosphate phosphatase/chronophin (PLPP/CIN) dephosphorylates and activates cofilin, which depolymerizes F-actin^5,28^. Recently, we found that PLPP/CIN dephosphorylates calsenilin (CSEN) and reversely regulates CSEN bindings to Kv4.2 (an A-type K^+^ channel) and *N*-methyl-D-aspartate receptor independent of cofilin-mediated F-actin dynamics. Furthermore, PLPP/CIN deletion exhibits the lower intensity (severity)^29^, duration, and progression of seizures, but the shorter latency of seizure onset in response to KA. PLPP/CIN overexpression reverses these phenomena^30^. These novel properties of PLPP/CIN provide the possibility that interaction of PLPP/CIN with other biological molecules may modulate neuronal hyperexcitability independent of F-actin depolymerization.

Here, we demonstrate that PLPP/CIN bound to NEDD4-2 and dephosphorylated its S448 site, which accelerated NEDD4-2 ubiquitination, and subsequently enhanced the responsiveness of α‐amino‐3‐hydroxy‐5‐methyl‐4‐isoxazolepropionic acid (AMPA) receptor by inhibiting NEDD4-2-mediated ubiquitination of GluA1 subunit, but not voltage-gated K^+^ channels KCNQ2/3/5. We further describe that NEDD4-2 knockdown increased seizure severity and facilitated its progression in response to KA, like PLPP/CIN overexpression. These findings indicate the novel function of PLPP/CIN as a NEDD4-2 phosphatase, which may regulate neuronal excitability by an inhibitory mechanism for E3 ubiquitin ligase activity of NEDD4-2. Therefore, we suggest that PLPP/CIN may be a potential therapeutic target for epileptogenesis and NEDD4-2-associated diseases.

## Results

### PLPP/CIN dephosphorylates NEDD4-2 at S448 site and accelerates NEDD4-2 ubiquitination

**Fig. 1:**
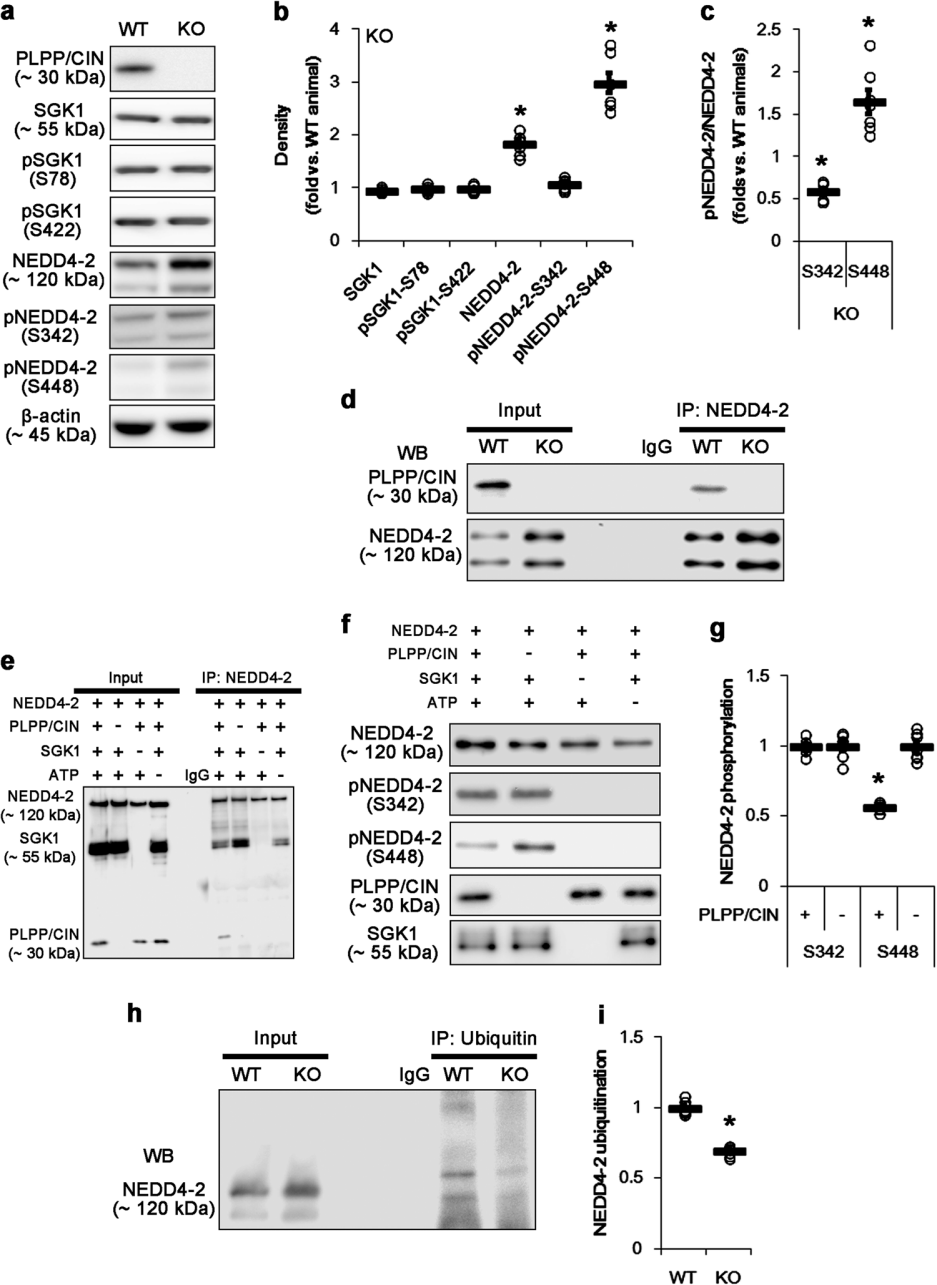
PLPP/CIN-mediated NEDD4-2 dephosphorylation. **a**–**c** Effect of PLPP/CIN deletion on expression and phosphorylation levels of SGK1 and NEDD4-2 in vivo. Total NEDD4-2 protein and pNEDD4-2 S448 phosphorylation levels are higher in *PLPP/CIN*^*−/−*^ mice, as compared to WT mice. **a** Representative western blots of SGK1 and NEDD4-2. **b**, **c** Quantification of SGK1 and NEDD4-2 expression, their phosphorylation levels, and the ratio of pNEDD4-2 to total NEDD4-2. Open circles indicate each individual value. Horizontal bars indicate mean value (mean ± S.E.M.; *p < 0.05 vs. WT animal; *n* = 7, respectively). **d** Representative co-immunoprecipitation data revealing PLPP/CIN-NEDD4-2 bindings. **e**–**g** In vitro assay using recombinant proteins. **e**, **f** Representative co-immunoprecipitation and western blot data demonstrating PLPP/CIN-NEDD4-2 bindings, and PLPP/CIN-mediated NEDD4-2 S448 dephosphorylation. **g** Quantification of NEDD4-2 phosphorylation levels based on western blot data. Open circles indicate each individual value. Horizontal bars indicate mean value (mean ± S.E.M.; *p < 0.05 vs. vehicle; *n* = 7). **h**, **i** Effect of PLPP/CIN deletion on NEDD4-2 ubiquitination in vivo. Co-immunoprecipitation reveals that PLPP/CIN deletion decreases the ubiquitin-NEDD4-2 bindings. **h** Representative western blots of ubiquitin-NEDD4-2 bindings. **i** Quantification of NEDD4-2 ubiquitination based on western blot data. Open circles indicate each individual value. Horizontal bars indicate mean value (mean ± S.E.M.; *p < 0.05 vs. WT animal; *n* = 7)

First, we validated the identification of PLPP/CIN as a NEDD4-2 phosphatase. PLPP/CIN knockout (*PLPP/CIN*^*−/−*^) mice showed the increases in NEDD4-2 protein and S448 phosphorylation levels to 1.8-fold and 3-fold of WT mice levels, respectively (*p* < 0.05 vs. WT mice, respectively; Fig. 1a–c), although S342 phosphorylation level was similar to that of WT mice. The ratio of S448 phosphorylation to total NEDD4-2 protein (S448 phosphorylation ratio) in *PLPP/CIN*^*−/−*^ mice was ~1.5-fold of WT mice level, while that of S342 phosphorylation to total NEDD4-2 protein (S342 phosphorylation ratio) was ~0.6-fold of WT mice level (*p* < 0.05 vs. WT mice, respectively; Fig. 1a, c). Since SGK1 directly phosphorylates NEDD4-2^22,26,27^, we explored whether PLPP/CIN regulates SGK1 activity. However, PLPP/CIN deletion did not influence SGK1 protein level and its phosphorylations on S78 or S422 sites (Fig. 1a, b), which are required for SGK1 activation^31^. Furthermore, PLPP/CIN bound to NEDD4-2 in WT mice in vivo (Fig. 1d). In vitro assay also revealed that PLPP/CIN bound to NEDD4-2 when SGK1 and ATP were present (Fig. 1e), and reduced S448 phosphorylation to ~50% of PLPP/CIN-omitted condition (*p* < 0.05, respectively; Fig. 1f, g). However, PLPP/CIN did not affect NEDD4-2 S342 phosphorylation. Therefore, our findings indicate that PLPP/CIN may bind to NEDD4-2 and dephosphorylate its S448 site.

Since phosphorylations regulate NEDD4-2 protein stability by inhibiting its ubiquitination^12,32,33^, the increased NEDD4-2 protein level induced by PLPP/CIN deletion suggests that NEDD4-2 S448 dephosphorylation may most likely affect ubiquitination of NEDD4-2. Thus, we investigated the effect of PLPP/CIN deletion on ubiquitin-NEDD4-2 binding in vivo. *PLPP/CIN*^*−/−*^ mice showed ~30% reduction of the binding of ubiquitin to NEDD4-2 (*p* < 0.05 vs. WT mice, respectively; Fig. 1h, i). These findings indicate that PLPP/CIN-mediated NEDD4-2 S448 dephosphorylation may elicit its ubiquitination under physiological condition.

### PLPP/CIN deletion attenuates seizure-induced NEDD4-2 ubiquitination

**Fig. 2:**
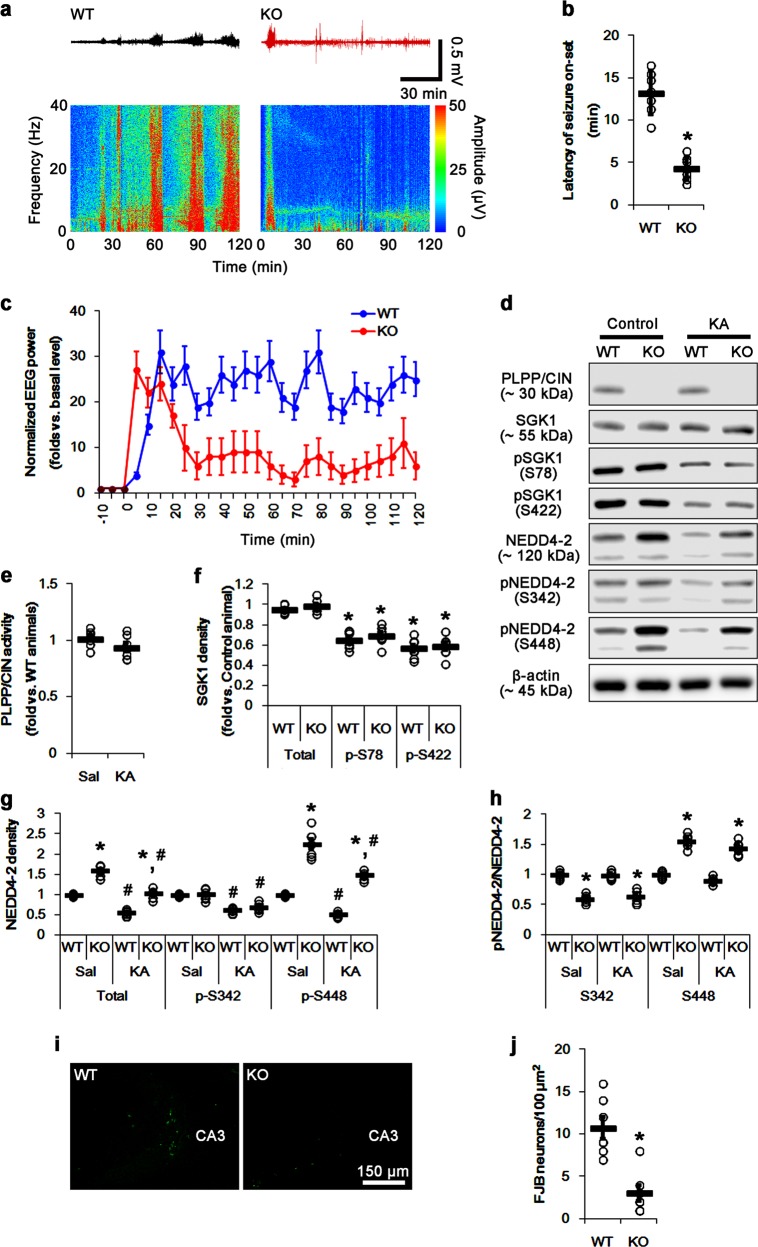
Effects of PLPP/CIN deletion on seizure activity and NEDD4-2 phosphorylations in response to KA. **a**–**c** Effect of PLPP/CIN deletion on seizure activity in response to KA. *PLPP/CIN*^*−/−*^ mice demonstrate the reductions in the latency of seizure onset, seizure intensity, and its duration. **a** Representative EEG traces and frequency-power spectral temporal maps in response to KA. **b** Quantification of latency of seizure onset. Open circles indicate each individual value. Horizontal bars indicate mean value (mean ± S.E.M.; *p < 0.05 vs. WT animals; *n* = 7, respectively). **c** Quantification of total EEG power (seizure intensity) in response to KA (mean ± S.E.M.; *n* = 7, respectively). **d**–**h** Effects of KA on PLPP/CIN activity, SGK1, and NEDD4-2 expressions and their phosphorylation levels. KA does not affect PLPP/CIN activity. PLPP/CIN deletion attenuates the reductions in total NEDD4-2 protein and pNEDD4-2 S448 phosphorylation levels, but not SGK1 and its phosphorylations, following KA injection. **d** Representative western blots of SGK1 and NEDD4-2. **e** Quantification of PLPP/CIN activity (*p < 0.05 vs. control WT animals; *n* = 7, respectively). **f** Quantification of SGK1 expression and its phosphorylation levels. Open circles indicate each individual value. Horizontal bars indicate mean value (mean ± S.E.M.; *p < 0.05 vs. control animals; *n* = 7, respectively). **g**, **h** Quantification of NEDD4-2 expression, its phosphorylation levels and the ratios of pNEDD4-2 to total NEDD4-2. Open circles indicate each individual value. Horizontal bars indicate mean value (mean ± S.E.M.; *^,*#*^*p* < 0.05 vs. WT and saline-treated animals, respectively; *n* = 7, respectively). **i**, **j** Effect of PLPP/CIN deletion on KA-induced neuronal death. PLPP/CIN deletion mitigates neuronal damage 1 day after KA injection. **i** Representative photos of FJB-positive degenerating neurons. **j** Quantification of the number of FJB-positive neurons in response to KA. Open circles indicate each individual value. Horizontal bars indicate mean value (mean ± S.E.M.; *p < 0.05 vs. WT animals; *n* = 7, respectively)

Recently, we have reported that *PLPP/CIN*^*−/−*^ mice show the shorter latency of seizure onset, but interrupt seizure progression in response to KA^30^. In contrast, spontaneous NEDD mutation in NEDD4-2^*andi*^ mice increases seizure susceptibility in response to KA^14^. Since 2h after KA injection is the suitable time point to compare the time of seizure onset, total EEG power and the changes in anatomical or biochemical profiles in *PLPP/CIN*^*Tg*^ and *PLPP/CIN*^*−/−*^ mice^30^, we investigated whether PLPP/CIN-mediated NEDD4-2 ubiquitination reciprocally regulates seizure activity 2 h after KA injection. Consistent with our previous report^30^, PLPP/CIN deletion reduced the latency of seizure onset, seizure intensity (severity), and seizure duration in response to KA (*p* < 0.05 vs. WT mice, respectively; Fig. 2a–c). After 2 h of KA injection, SGK1 phosphorylations were diminished in WT mice without changing its protein level and PLPP/CIN activity (*p* < 0.05 vs. control animals; Fig. 2d–f). NEDD4-2 protein level and its phosphorylations were decreased to ~0.6-fold of control level (*p* < 0.05 vs. control animals; Fig. 2d, g). KA also decreased SGK1 phosphorylations in *PLPP/CIN*^*−/−*^ mice without altering its protein level (*p* < 0.05 vs. control animals; Fig. 2d, f). However, PLPP/CIN deletion attenuated the reductions in NEDD4-2 protein and S448 (not S342) phosphorylation levels (*p* < 0.05 vs. WT mice; Fig. 2d, g). Because of the reduced NEDD4-2 protein level, the phosphorylation ratios of S342 and S448 in WT mice were similar to those in WT control animals following KA injection (Fig. 2d, h). In *PLPP/CIN*^*−/−*^ mice, S342 phosphorylation ratio was lower than that in WT mice, while S448 phosphorylation ratio was higher than WT mice (*p* < 0.05 vs. WT mice, respectively; Fig. 2d, h). Consistent with seizure severity, PLPP/CIN deletion attenuated seizure-induced neuronal damage in the CA3 region (*p* < 0.05 vs. WT mice; Fig. 2i, j). These findings indicate that PLPP/CIN may reduce NEDD4-2 protein level and its S448 phosphorylation induced by seizure activity independent of SGK1 activity, and that upregulated NEDD4-2 protein level in *PLPP/CIN*^*−/−*^ mice may abrogate seizure progression in response to KA.

**Fig. 3:**
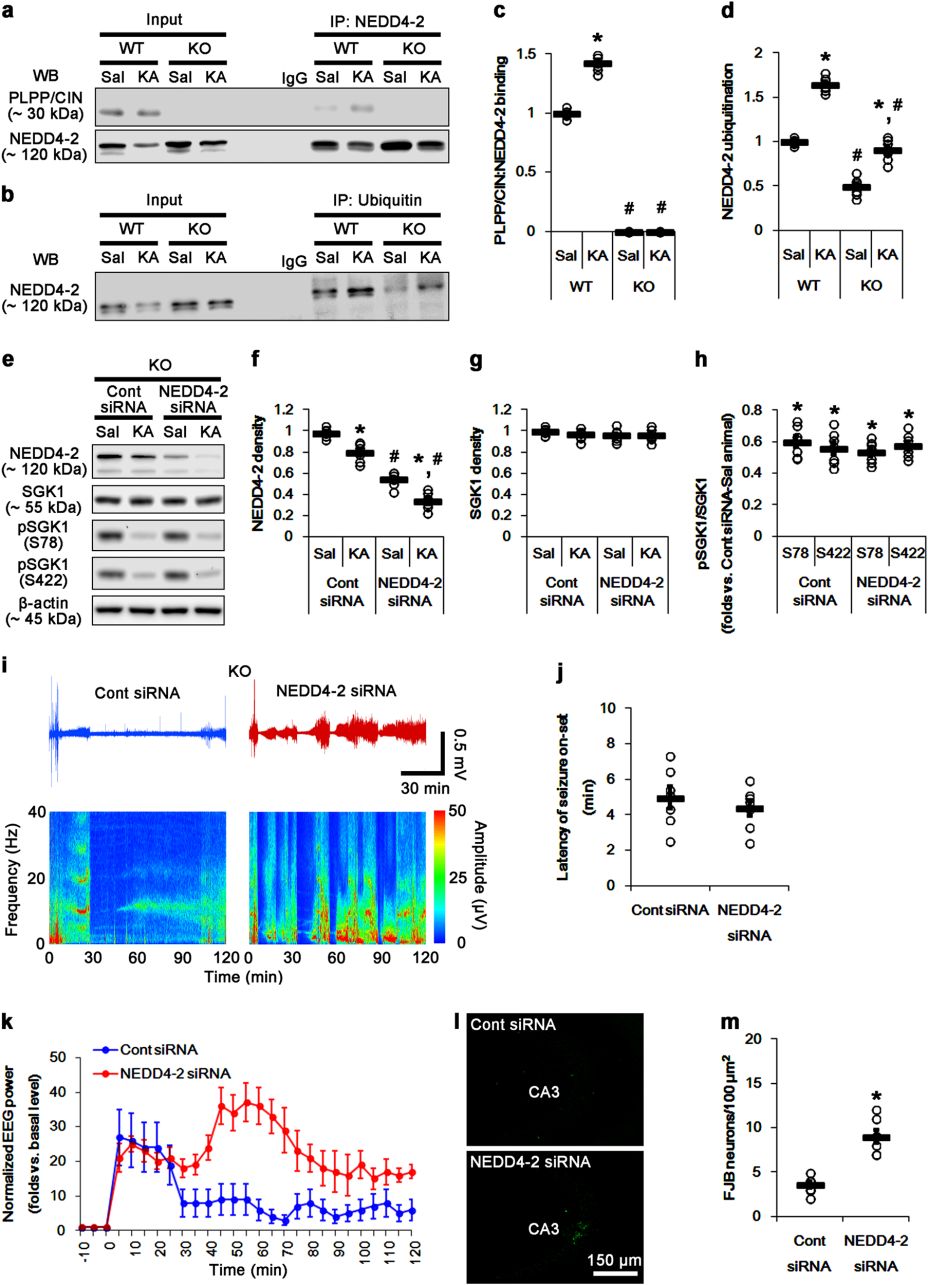
Effect of PLPP/CIN deletion on NEDD4-2 ubiquitination and that of NEDD4-2 knockdown on seizure activity in response to KA in *PLPP/CIN*^*−/−*^ mice. **a**–**d** Effect of PLPP/CIN-NEDD4-2 binding on NEDD4-2 ubiquitination following KA injection. PLPP/CIN deletion attenuates NEDD4-2 ubiquitination induced by KA. **a**, **b** Representative western blots of PLPP/CIN- and ubiquitin-NEDD4-2 bindings. **c**, **d** Quantification of PLPP/CIN-NEDD4-2 bindings and NEDD4-2 ubiquitination based on western blot data. Open circles indicate each individual value. Horizontal bars indicate mean value (mean ± S.E.M.; *^,*#*^*p* < 0.05 vs. saline-treated and WT animals, respectively; *n* = 7). **e**–**h** Effects of NEDD4-2 knockdown on NEDD4-2 expression, and SGK1 expression/phosphorylation in response to KA in *PLPP/CIN*^*−/−*^ mice. NEDD4-2 knockdown reduces total NEDD4-2 protein level, but not SGK1 and its phosphorylations, in *PLPP/CIN*^*−/−*^ mice following KA injection. **e** Representative western blots of SGK1 and NEDD4-2. **f**–**h** Quantification of NEDD4-2 expression and SGK1 expression/phosphorylation levels. Open circles indicate each individual value. Horizontal bars indicate mean value (mean ± S.E.M.; *^,*#*^*p* < 0.05 vs. saline- and control siRNA-treated animals, respectively; *n* = 7, respectively). **i**–**k** Effects of NEDD4-2 knockdown on seizure activity in response to KA in *PLPP/CIN*^*−/−*^ mice. NEDD4-2 knockdown increases seizure intensity and its duration in *PLPP/CIN*^*−/−*^ mice. **i** Representative EEG traces and frequency-power spectral temporal maps in response to KA. **j** Quantification of latency of seizure onset. Open circles indicate each individual value. Horizontal bars indicate mean value (*n* = 7, respectively). **k** Quantification of total EEG power (seizure intensity) in response to KA (mean ± S.E.M.; *n* = 7, respectively). **l**, **m** The effect of NEDD4-2 knockdown on KA-induced neuronal death in *PLPP/CIN*^*−/−*^ mice. NEDD4-2 siRNA exacerbates neuronal damage in *PLPP/CIN*^*−/−*^ mice 1 day after KA injection. **l** Representative photos of FJB-positive degenerating neurons. **m** Quantification of the number of FJB-positive neurons in response to KA. Open circles indicate each individual value. Horizontal bars indicate mean value (mean ± S.E.M.; *p < 0.05 vs. control siRNA; *n* = 7, respectively)

Next, we explored whether seizure activity affects the binding of PLPP/CIN to NEDD4-2 and the NEDD4-2 ubiquitination. Under physiological condition, the NEDD4-2 ubiquitination in *PLPP/CIN*^*−/−*^ mice was ~0.5-fold of WT mice level (*p* < 0.05 vs. WT mice; Fig. 3a–d). KA increased the PLPP/CIN-NEDD4-2 binding and NEDD4-2 ubiquitination to ~1.4-fold and ~1.6-fold of control level in WT mice (*p* < 0.05 vs. control animals; Fig. 3a–d). KA-induced NEDD4-2 ubiquitination in *PLPP/CIN*^*−/−*^ mice was lower than that in WT mice (*p* < 0.05 vs. WT mice; Fig. 3a–d). These findings suggest that PLPP/CIN-mediated S448 dephosphorylation may play an important role in NEDD4-2 ubiquitination, which would regulate seizure progression.

### NEDD4-2 knockdown increases the latency of seizure onset and its seizure severity in *PLPP/CIN*^*−/−*^ mice following KA injection

To confirm the role of PLPP/CIN-mediated NEDD4-2 ubiquitination in seizure activity, we applied NEDD4-2 siRNA in *PLPP/CIN*^*−/−*^ mice prior to KA injection. NEDD4-2 siRNA diminished NEDD4-2 protein level (*p* < 0.05 vs. control siRNA; Fig. 3e, f), while it did not affect SGK1 expression and its phosphorylation levels (Fig. 3e, g, h). NEDD4-2 knockdown did not affect the latency of seizure onset and seizure intensity in *PLPP/CIN*^*−/−*^ mice that were observed at 5–30 min after KA injection (Fig. 3i–k). However, NEDD4-2 siRNA produced the seizure progression in *PLPP/CIN*^*−/−*^ mice at 30–120 min after KA injection (*p* < 0.05 vs. control siRNA; Fig. 3i–k). After 2 h of KA injection, NEDD4-2 knockdown reinforced the reduced NEDD4-2 protein level (*p* < 0.05 vs. control siRNA; Fig. 3e, f). In addition, NEDD4-2 knockdown exacerbated seizure-induced CA3 neuronal damage in *PLPP/CIN*^*−/−*^ mice (*p* < 0.05 vs. control siRNA; Fig. 3l, m). These findings indicate that the increased NEDD4-2 protein level induced by PLPP/CIN deletion may inhibit the seizure progression in response to KA.

### PLPP/CIN deletion reduces membrane GluA1, but not KCNQ channels, expression

**Fig. 4:**
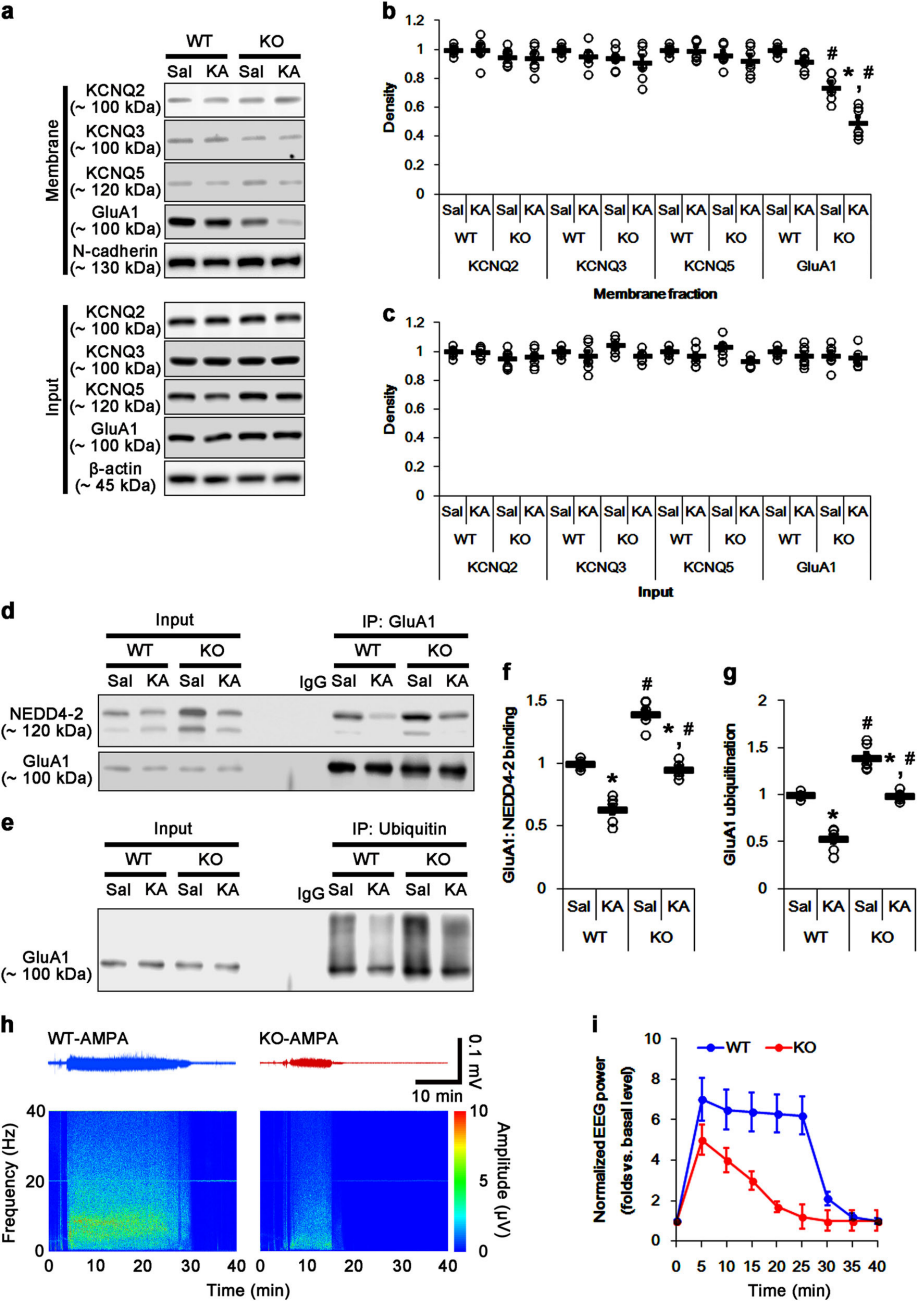
Effects of PLPP/CIN deletion on surface KCNQ and GluA1 expression, GluA1-NEDD4-2 binding, GluA1 ubiquitination, and neuronal activity in response to AMPA. **a**–**c** Effects of PLPP/CIN deletion on surface KCNQ and GluA1 expression following KA injection. As compared to WT mice, PLPP/CIN^*−/−*^ mice show the diminished surface GluA1, but not KCNQ2/3/5, expression under physiological condition and after KA injection. **a** Representative western blots of surface and total KCNQ and GluA1 expressions. **b**, **c** Quantification of surface and total KCNQ and GluA1 expressions based on western blot data. Open circles indicate each individual value. Horizontal bars indicate mean value (mean ± S.E.M.; *^,*#*^*p* < 0.05 vs. saline-treated and WT animals, respectively; *n* = 7). **d**–**g** Effects of PLPP/CIN deletion on GluA1-NEDD4-2 binding and GluA1 ubiquitination. As compared to WT mice, *PLPP/CIN*^*−/−*^ mice show the increased GluA1-NEDD4-2 bindings and GluA1 ubiquitination under physiological condition and after KA injection. **d**, **e** Representative western blots of GluA1-NEDD4-2 binding and GluA1 ubiquitination. **f**, **g** Quantification of GluA1-NEDD4-2 binding and GluA1 ubiquitination based on western blot data. Open circles indicate each individual value. Horizontal bars indicate mean value (mean ± S.E.M.; *^,*#*^*p* < 0.05 vs. saline-treated and WT animals, respectively; *n* = 7). **h**, **i** Effect of PLPP/CIN deletion on neuronal activity in response to AMPA. *PLPP/CIN*^*−/−*^ mice demonstrate the reduced neuronal activity in response to AMPA. **h** Representative EEG traces and frequency-power spectral temporal maps in response to AMPA. **i** Quantification of total EEG power in response to AMPA (mean ± S.E.M.; *n* = 7, respectively)

In the present study, NEDD4-2 siRNA propelled KA-induced seizure activity in *PLPP/CIN*^*−/−*^ mice. However, it is unknown what NEDD4-2 substrates that modulate seizure activity would be affected by PLPP/CIN deletion. NEDD4-2 regulates the generation of muscarine-sensitive K^+^ current (M-current), which is required for stabilizing the resting membrane potential and limiting neuronal excitability, by ubiquitinating KCNQ2/3/5 channels^34^. Indeed, mutations in KCNQ are associated with various epilepsy phenotypes including benign familial neonatal seizures^35–37^. Therefore, it is likely that PLPP/CIN-mediated NEDD4-2 ubiquitination would affect KA-induced seizure activity by altering surface KCNQ2/3/5 expressions. To elucidate this hypothesis, we explored the membrane expression levels of KCNQ2/3/5 in *PLPP/CIN*^*−/−*^ mice. However, there was no differences in total and membrane KCNQ2/3/5 expressions between WT and *PLPP/CIN*^*−/−*^ mice, which were unaffected by KA injection (Fig. 4a–c). These findings indicate that KCNQ2/3/5 ubiquitination may not be involved in the regulation of PLPP/CIN-mediated seizure progression.

On the other hand, NEDD4-2 plays a role in neuronal activity and seizure susceptibility through GluA1 ubiquitination, which is facilitated by its phosphorylations^24,38^. Indeed, NEDD4-2^*andi*^ mice show the increases in total and membrane GluA1 expression levels and seizure susceptibility in response to KA^14^. Since PLPP/CIN deletion impairs the bidirectional synaptic plasticity (long-term potentiation and long-term depression), which is mediated by AMPAR^28^, we accessed whether PLPP/CIN deletion affects NEDD4-2-mediated GluA1 ubiquitination. Consistent with our previous study^28^, there was no difference in total GluA1 protein level between WT and *PLPP/CIN*^*−/−*^ mice (Fig. 4a, c). However, *PLPP/CIN*^*−/−*^ mice showed the reduced membrane GluA1 expression concomitant with the increased GluA1-NEDD4-2 binding and GluA1 ubiquitination (*p* < 0.05 vs. WT mice, Fig. 4a–g). KA did not affect the surface GluA1 expression in WT mice (Fig. 4a, b), accompanied by reducing GluA1-NEDD4-2 binding and GluA1 ubiquitination (*p* < 0.05 vs. control animals, Fig. 4d–g). In *PLPP/CIN*^*−/−*^ mice, KA also reduced membrane GluA1 expression, GluA1-NEDD4-2 binding and GluA1 ubiquitination (*p* < 0.05 vs. control animals, Fig. 4a–g). However, the levels of GluA1-NEDD4-2 binding and GluA1 ubiquitination were higher in *PLPP/CIN*^*−/−*^ mice than those in WT mice 2 h after KA injection (*p* < 0.05 vs. WT mice, Fig. 4d–g). Total GluA1 protein level was unaffected by KA in both WT and *PLPP/CIN*^*−/−*^ mice (Fig. 4a, c). To confirm the NEDD4-2-mediated neuronal excitability via regulation of membrane GluA1 expression in *PLPP/CIN*^*−/−*^ mice, we applied focal AMPA injection (20 μM) into the dentate gyrus^29^. As compared to WT mice, the efficacy of AMPA-mediated neuronal discharge was lower in *PLPP/CIN*^*−/−*^ mice (*p* < 0.05 vs. WT mice, Fig. 4h, i). These data indicate that the increased NEDD4-2 protein in *PLPP/CIN*^*−/−*^ mice may contribute to the abrogation of seizure progression by inhibiting GluA1 functionality via ubiquitination.

### NEDD4-2 knockdown ameliorates GluA1 ubiquitination in *PLPP/CIN*^*−/−*^ mice

**Fig. 5:**
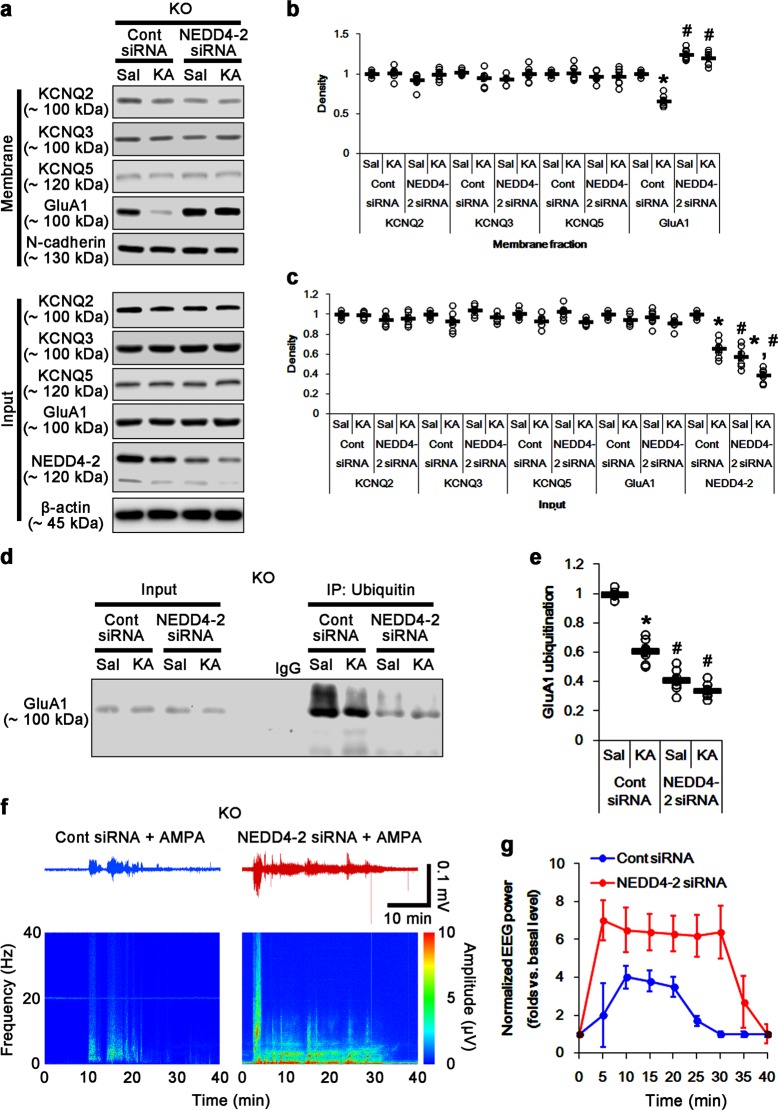
Effects of NEDD4-2 knockdown on surface KCNQ and GluA1 expression, GluA1-NEDD4-2 bindings, GluA1 ubiquitination, and neuronal activity in response to AMPA in *PLPP/CIN*^*−/−*^ mice. **a**–**c** Effects of NEDD4-2 knockdown on surface KCNQ and GluA1 expression following KA injection in *PLPP/CIN*^*−/−*^ mice. As compared to control siRNA, NEDD4-2 siRNA increases surface GluA1, but not KCNQ2/3/5, expression in *PLPP/CIN*^*−/−*^ mice under physiological condition and after KA injection. **a** Representative western blots of surface and total KCNQ and GluA1 expressions. **b**, **c** Quantification of surface and total KCNQ and GluA1 expressions based on western blot data. Open circles indicate each individual value. Horizontal bars indicate mean value (mean ± S.E.M.; *^,*#*^*p* < 0.05 vs. saline- and control siRNA-treated animals, respectively; *n* = 7). **d**, **e** Effects of NEDD4-2 siRNA on GluA1 ubiquitination in *PLPP/CIN*^*−/−*^ mice. NEDD4-2 knockdown reduces GluA1-NEDD4-2 bindings and GluA1 ubiquitination in *PLPP/CIN*^*−/−*^ mice under physiological condition and after KA injection. **d** Representative western blots of GluA1 ubiquitination. **e** Quantification of GluA1 ubiquitination based on western blot data. Open circles indicate each individual value. Horizontal bars indicate mean value (mean ± S.E.M.; *^,*#*^*p* < 0.05 vs. saline- and control siRNA-treated animals, respectively; *n* = 7). **f**, **g** Effects of NEDD4-2 siRNA on neuronal activity in response to AMPA in *PLPP/CIN*^*−/−*^ mice. NEDD4-2 knockdown increases the neuronal activity in response to AMPA in *PLPP/CIN*^*−/−*^ mice. **f** Representative EEG traces and frequency-power spectral temporal maps in response to AMPA. **g** Quantification of total EEG power in response to AMPA (mean ± S.E.M.; *n* = 7, respectively)

To directly evaluate the role of NEDD4-2 in GluA1 ubiquitination and its surface expression, we applied NEDD4-2 knockdown in *PLPP/CIN*^*−/−*^ mice. NEDD4-2 siRNA increased surface GluA1 expression, and decreased GluA1 ubiquitination in *PLPP/CIN*^*−/−*^ mice without altering total GluA1 protein level (*p* < 0.05 vs. control siRNA; Fig. 5a–e). KA did not influence the changes in surface GluA1 expression and GluA1 ubiquitination induce by NEDD4-2 knockdown (*p* < 0.05 vs. control siRNA; Fig. 5a–e). Furthermore, NEDD4-2 siRNA increased the efficacy of AMPA-dependent neuronal discharge (*p* < 0.05 vs. control siRNA, Fig. 5f–g). These findings indicate that the upregulated NEDD4-2 protein in *PLPP/CIN*^*−/−*^ mice may directly reduce spontaneous GluA1-mediated neuronal activity. However, NEDD4-2 siRNA did not affect total protein levels and membrane expressions of KCNQ2/3/5 in control- and KA-injected *PLPP/CIN*^*−/−*^ mice (Fig. 5a–c), which reveal the role of NEDD4-2 in limiting GluA1 surface expression in the present study. Thus, our findings suggest that PLPP/CIN deletion may reduce surface GluA1 expression by facilitating NEDD4-2-mediated GluA1 ubiquitination.

### PLPP/CIN overexpression increases NEDD4-2 ubiquitination and facilitates seizure progression induced by KA

**Fig. 6:**
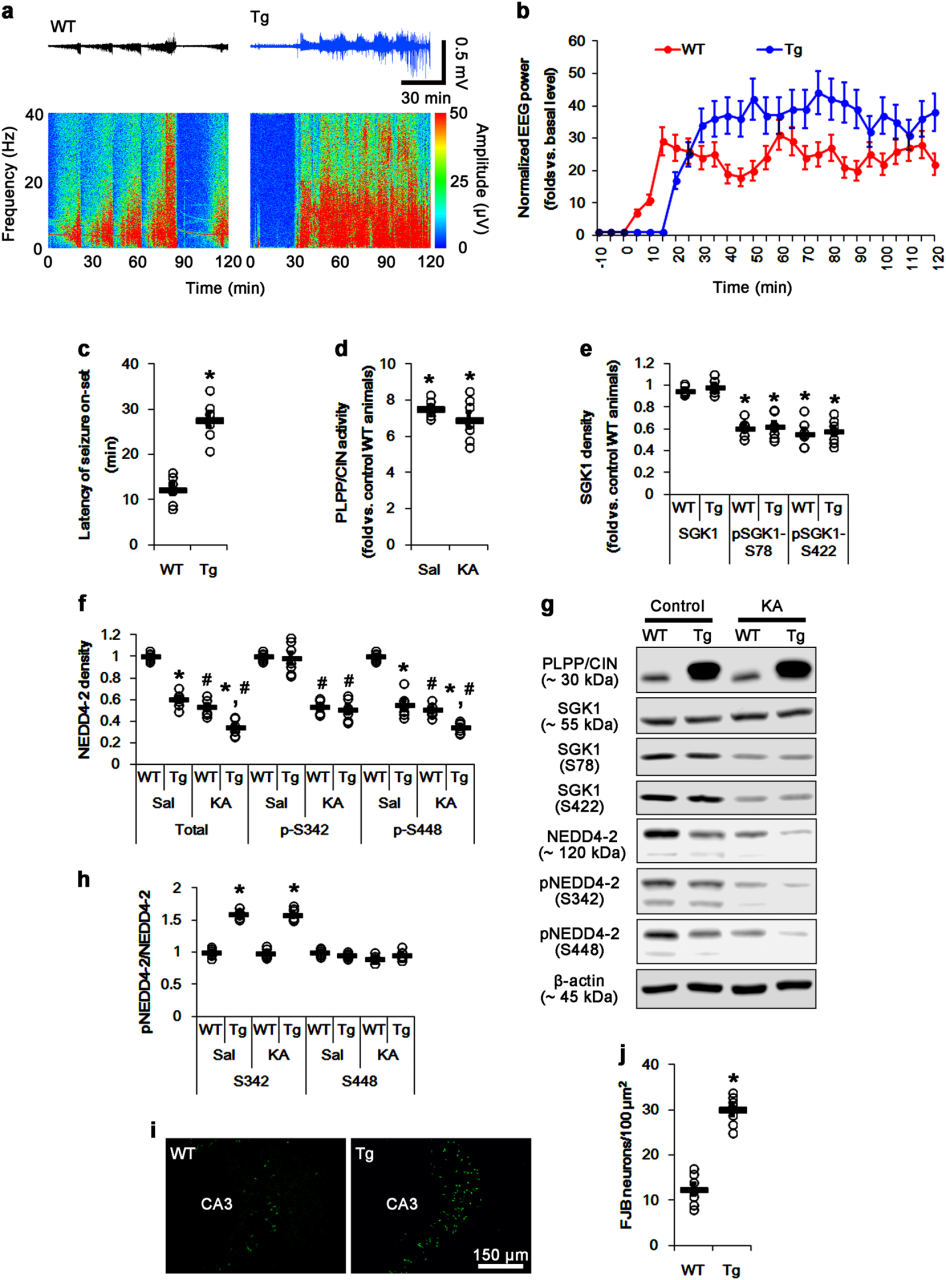
Effects of PLPP/CIN overexpression on seizure activity and NEDD4-2 phosphorylations in response to KA. **a**–**c** Effect of PLPP/CIN overexpression on seizure activity in response to KA. *PLPP/CIN*^*Tg*^ mice demonstrate the increases in the latency of seizure onset and seizure intensity. **a** Representative EEG traces and frequency-power spectral temporal maps in response to KA. **b** Quantification of total EEG power in response to KA (mean ± S.E.M.; *n* = 7, respectively). **c** Quantification of the latency of seizure onset. Open circles indicate each individual value. Horizontal bars indicate mean value (mean ± S.E.M.; *p < 0.05 vs. WT animals; *n* = 7, respectively). **d**–**h** Effects of PLPP/CIN overexpression on PLPP/CIN activity, SGK1, and NEDD4-2 phosphorylations in response to KA. PLPP/CIN overexpression increases PLPP/CIN activity under physiological condition, and facilitates the reductions in total NEDD4-2 and pNEDD4-2 S448 phosphorylation levels, but not SGK1 and its phosphorylations, under physiological condition and after KA injection. **d** Quantification of PLPP/CIN activity (*p < 0.05 vs. control WT animals; *n* = 7, respectively). **e** Quantification of SGK1 and its phosphorylation levels (*p < 0.05 vs. control animals; *n* = 7, respectively). **f** Quantification of NEDD4-2 expression and its phosphorylation levels. Open circles indicate each individual value. Horizontal bars indicate mean value (mean ± S.E.M.; *^,*#*^*p* < 0.05 vs. WT and saline-treated animals; *n* = 7, respectively). **g** Representative western blots of SGK1 and NEDD4-2. **h** Quantification of the ratios of pNEDD4-2 to total NEDD4-2. Open circles indicate each individual value. Horizontal bars indicate mean value (mean ± S.E.M.; *p < 0.05 vs. WT animals, respectively; *n* = 7, respectively). **i**, **j** Effect of PLPP/CIN overexpression on KA-induced neuronal death. PLPP/CIN overexpression aggravates neuronal damage 1 day after KA injection. **i** Representative photos of FJB-positive degenerating neurons. **j** Quantification of the number of FJB-positive neurons in response to KA. Open circles indicate each individual value. Horizontal bars indicate mean value (mean ± S.E.M.; *p < 0.05 vs. WT animals; *n* = 7, respectively)

To further elucidate the role of PLPP/CIN in NEDD4-2-mediated GluA1 ubiquitination, we employed PLPP/CIN transgenic (*PLPP/CIN*^*Tg*^) mice. Consistent with our previous study^30^, *PLPP/CIN*^*Tg*^ mice showed the increases in the seizure latency and seizure intensity (severity) in response to KA (*p* < 0.05 vs. WT mice, respectively; Fig. 6a–c). Under physiological condition, the PLPP/CIN activity in *PLPP/CIN*^*Tg*^ mice was 7.5-fold higher than that in WT mice under physiological condition (*p* < 0.05 vs. WT mice, Fig. 6d). *PLPP/CIN*^*Tg*^ mice also demonstrated the reductions in NEDD4-2 protein and S448 (not S342) phosphorylation levels without altering SGK1 activity (*p* < 0.05 vs. WT mice, respectively; Fig. 6e–g). Since *PLPP/CIN*^*Tg*^ mice showed the downregulation of NEDD4-2 protein level (*p* < 0.05 vs. WT mice, Fig. 6f–g), S342 phosphorylation ratio in *PLPP/CIN*^*Tg*^ mice was higher than that in WT mice, but the S448 phosphorylation ratio in *PLPP/CIN*^*Tg*^ mice was similar to that in WT mice (*p* < 0.05 vs. WT mice, respectively; Fig. 6g, h). After 2 h of KA injection, PLPP/CIN activity was slightly decreased, but not significantly, in *PLPP/CIN*^*Tg*^ mice (Fig. 6d). SGK1 phosphorylations were decreased to 0.5-fold–0.6-fold of control level in both WT and *PLPP/CIN*^*Tg*^ mice without changing its expression level (*p* < 0.05 vs. control animals, respectively; Fig. 6e, g). There was no difference in the altered SGK1 phosphorylations between both groups (Fig. 6e, g). KA decreased NEDD4-2 expression and its phosphorylations to about half-fold of control level in both WT and *PLPP/CIN*^*Tg*^ mice (*p* < 0.05 vs. control animals; Fig. 6f, g). KA did not affect the S342 and S448 phosphorylation ratios in both groups (Fig. 6g, h). PLPP/CIN overexpression aggravated KA-induced neuronal damage in the CA3 region (*p* < 0.05 vs. WT mice; Fig. 6i, j).

**Fig. 7:**
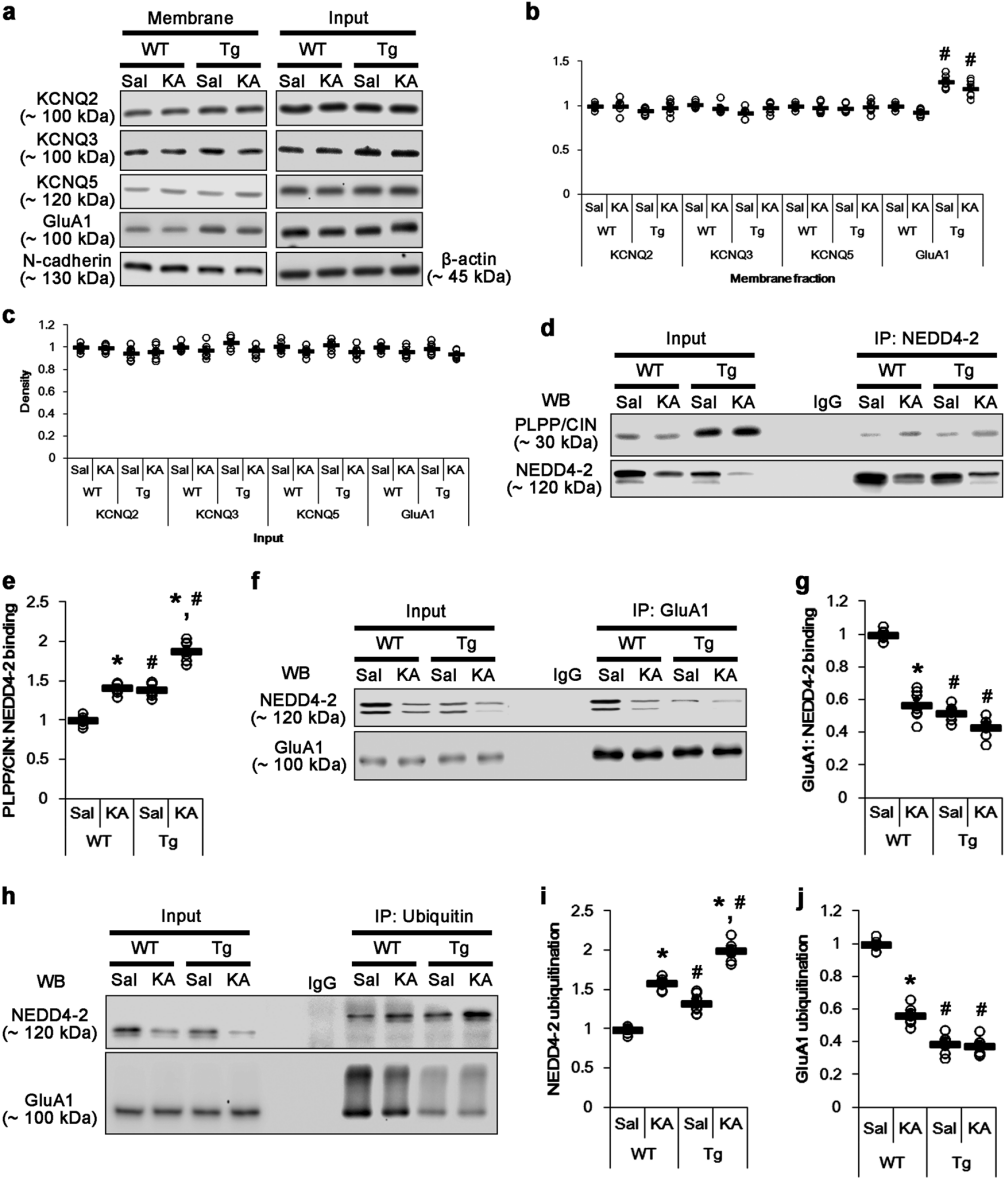
Effects of PLPP/CIN overexpression on surface KCNQ and GluA1 expression, GluA1-NEDD4-2 bindings, and GluA1 ubiquitination. **a**–**c** Effects of PLPP/CIN overexpression on surface KCNQ and GluA1 expression following KA injection. As compared to WT mice, *PLPP/CIN*^*Tg*^ mice demonstrate increases surface GluA1, but not KCNQ2/3/5, expression under physiological condition and after KA injection. **a** Representative western blots of surface and total KCNQ and GluA1 expressions. **b**, **c** Quantification of surface and total KCNQ and GluA1 expressions based on western blot data. Open circles indicate each individual value. Horizontal bars indicate mean value (mean ± S.E.M.; *^,*#*^*p* < 0.05 vs. saline-treated and WT animals, respectively; *n* = 7). **d**, **e** Effects of PLPP/CIN overexpression on PLPP/CIN-NEDD4-2 bindings. As compared to WT mice, *PLPP/CIN*^*Tg*^ mice show the increased PLPP/CIN-NEDD4-2 binding under physiological condition and after KA injection. **d** Representative western blots of PLPP/CIN-NEDD4-2 binding. **e** Quantification of PLPP/CIN-NEDD4-2 binding based on western blot data. Open circles indicate each individual value. Horizontal bars indicate mean value (mean ± S.E.M.; *^,*#*^*p* < 0.05 vs. saline-treated and WT animals, respectively; *n* = 7). **f**, **g** Effects of PLPP/CIN overexpression on GluA1-NEDD4-2 binding. *PLPP/CIN*^*Tg*^ mice demonstrate the diminished GluA1-NEDD4-2 binding under physiological condition and after KA injection. **f** Representative western blots of PLPP/CIN-NEDD4-2 bindings. **g** Quantification of PLPP/CIN-NEDD4-2 bindings based on western blot data. Open circles indicate each individual value. Horizontal bars indicate mean value (mean ± S.E.M.; *^,*#*^*p* < 0.05 vs. saline-treated and WT animals, respectively; *n* = 7). **h**–**j** Effects of PLPP/CIN overexpression on the ubiquitinations of NEDD4-2 and GluA1. *PLPP/CIN*^*Tg*^ mice demonstrate the increased NEDD4-2 ubiquitination, but the reduced GluA1 ubiquitination under physiological condition and after KA injection. **h** Representative western blots of PLPP/CIN-NEDD4-2 bindings. **i**, **j** Quantification of ubiquitinations of NEDD4-2 and GluA1 based on western blot data. Open circles indicate each individual value. Horizontal bars indicate mean value (mean ± S.E.M.; *^,*#*^*p* < 0.05 vs. saline-treated and WT animals, respectively; *n* = 7)

**Fig. 8:**
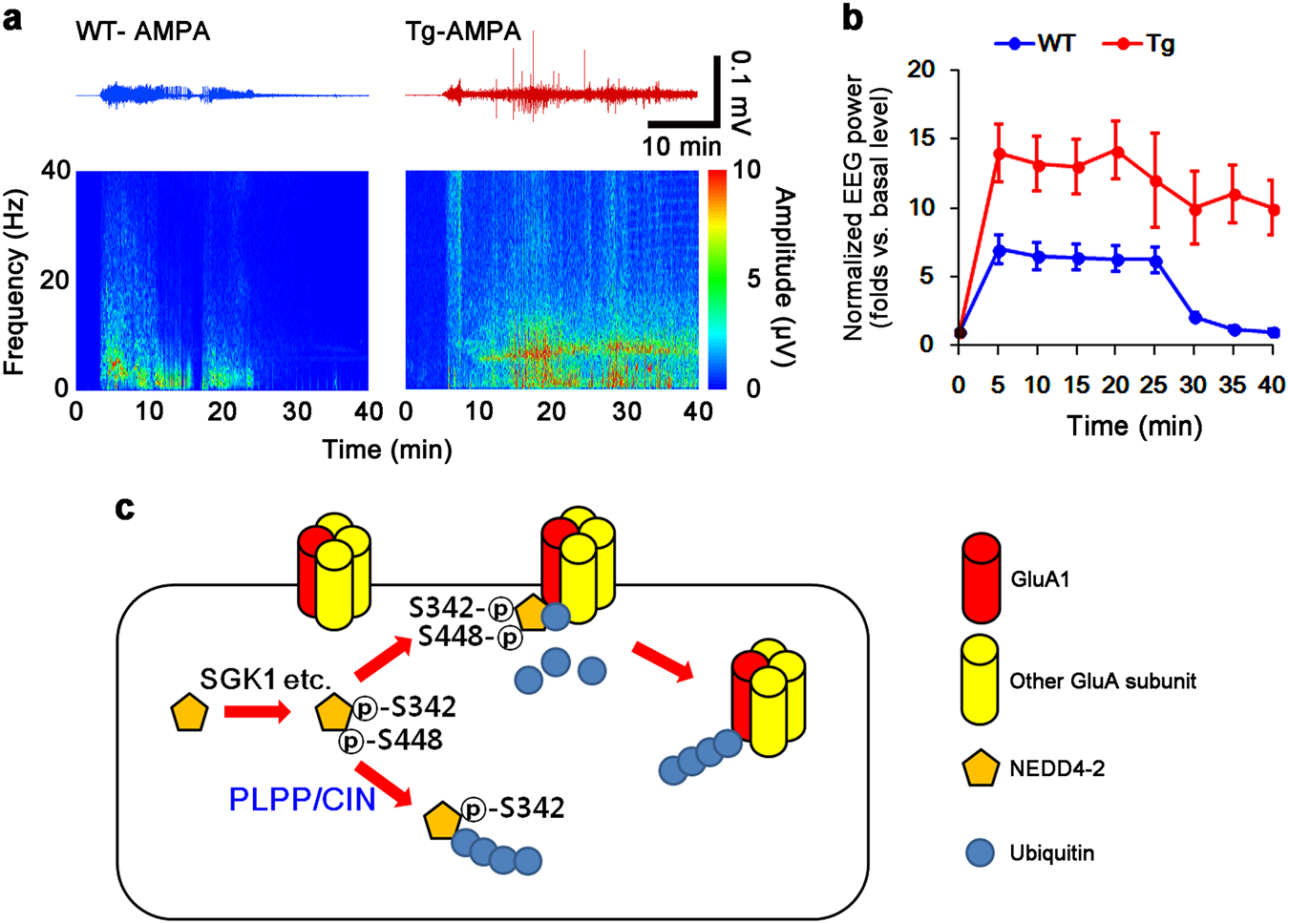
Effect of neuronal activity in response to AMPA and the hypothesized role of PCPP/CIN-mediated GluA1 ubiquitination. **a**, **b** Effects of PLPP/CIN overexpression on neuronal activity in response to AMPA. *PLPP/CIN*^*Tg*^ mice demonstrate the increased neuronal activity in response to AMPA. **a** Representative EEG traces and frequency-power spectral temporal maps in response to AMPA. **b** Quantification of total EEG power in response to AMPA (mean ± S.E.M.; *p* < 0.05 vs. WT animals; *n* = 7, respectively). **c** Scheme of inhibitory role of PLPP/CIN in NEDD4-2-mediated GluA1 ubiquitination. Protein kinases including SGK1 phosphorylate NEDD4-2 S342 and S448 sites, which facilitate its binding to and ubiquitination of GluA1. However, PLPP/CIN dephosphorylates NEDD4-2 S448 site, which leads to ubiquitination of NEDD4-2 and subsequently inhibits GluA1 ubiquitination, independent of SGK1 activity. These PLPP/CIN-mediated functional couplings of NEDD4-2 and GluA1 increase seizure intensity and its progression

*PLPP/CIN*^*Tg*^ mice did not show the changes in total protein levels and membrane expressions of KCNQ2/3/5 channels under physiological condition, as compared to WT mice (Fig. 7a–c). However, *PLPP/CIN*^*Tg*^ mice showed the enhanced membrane GluA1 expression without altering total GluA1 protein level (*p* < 0.05 vs. WT mice, Fig. 7a–c). The binding of NEDD4-2 to PLPP/CIN in *PLPP/CIN*^*Tg*^ mice was ~1.4-fold higher than that in WT mice (*p* < 0.05; Fig. 7d, e), while the GluA1-NEDD4-2 bindings was ~0.5-fold of WT mouse level (*p* < 0.05; Fig. 7f, g). The ubiquitination levels of NEDD4-2 and GluA1 in *PLPP/CIN*^*Tg*^ mice were 1.3-fold and 0.4-fold of WT mouse levels, respectively (*p* < 0.05; Fig. 7h–j). KA did not affect total and membrane KCNQ2/3/5 and GluA1 levels in both groups (Fig. 7a–c). However, KA increased PLPP/CIN-NEDD4-2 binding in both groups (*p* < 0.05 vs. control animals; Fig. 7d, e). Furthermore, KA decreased the GluA1-NEDD4-2 bindings in WT mice (*p* < 0.05 vs. control animals; Fig. 7f, g), but not in *PLPP/CIN*^*Tg*^ mice (Fig. 7f, g). KA increased NEDD4-2 ubiquitination in both groups (*p* < 0.05 vs. control animals; Fig. 7h, i). NEDD4-2 ubiquitination in *PLPP/CIN*^*Tg*^ mice was higher than that in WT mice (p < 0.05 vs. WT mice; Fig. 7h, i). In contrast, KA decreased GluA1 ubiquitination in WT mice, but not in *PLPP/CIN*^*Tg*^ mice (*p* < 0.05 vs. control animals; Fig. 7h, j). Focal AMPA injection revealed that the efficacy of AMPA-mediated neuronal discharge in *PLPP/CIN*^*Tg*^ mice was higher than that in WT mice (*p* < 0.05 vs. WT mice, Fig. 8a, b). These findings indicate that impaired NEDD4-2-mediated GluA1 ubiquitination may be responsible for seizure progression in *PLPP/CIN*^*Tg*^ mice. Taken together, our findings suggest that PLPP/CIN-mediated NEDD4-2 S448 dephosphorylation may facilitate NEDD4-2 ubiquitination, which may enhance surface AMPAR expression and seizure progression (Fig. 8c).

## Discussion

The novel findings in the present study are that PLPP/CIN dephosphorylates NEDD4-2 S448 site under physiological and postseizure conditions. NEDD4-2 phosphorylation increases surface expressions of KCNQ^34,39^, epithelial Na^+^ Channel (ENaC)^21,23^, and cardiac voltage-gated Na^+^ channel^40^. However, phosphorylations cannot completely suppress NEDD4-2 function^41^. Indeed, NEDD4-2 phosphorylation does not affect the ubiquitination of neuronal voltage-gated Na^+^ channels^19^. Furthermore, NEDD4-2 phosphorylation is required for a ENaC ubiquitination^23^. In the present study, we found that PLPP/CIN bound to NEDD4-2 and reduced S448 phosphorylation without changing NEDD4-2 S342 phosphorylation, and subsequently diminished NEDD4-2-mediated GluA1 ubiquitination. Since PLPP/CIN deletion and overexpression cannot influence the expression levels and activities of protein phosphatase 1A, protein phosphatase 2A, and protein phosphatase 2B under physiological condition and following KA injection^30^, our findings suggest that PLPP/CIN may specifically dephosphorylate S448 residue on NEDD4-2, and inhibit NEDD4-2 E3 ubiquitin ligase activity by reducing NEDD4-2 protein level.

Aforementioned, S448 site plays a role in NEDD4-2 protein stability independent of 14-3-3 bindings, since inhibition of NEDD4-2 S448 phosphorylation reduces NEDD4-2 protein level^12,25^. In the present study, PLPP/CIN deletion increased NEDD4-2 protein level with enhancing NEDD4-2 S448 phosphorylation and reducing NEDD4-2 ubiquitination under physiological condition and after KA injection, while PLPP/CIN overexpression showed the reverse effects. With respect to ubiquitination of NEDD4-2^32,33^, these findings suggest that PLPP/CIN-mediated NEDD4-2 S448 dephosphorylation may be one of inhibitory mechanisms for regulation of NEDD4-2 activity via its ubiquitination. In the present study, furthermore, PLPP/CIN deletion reduced the phosphorylation ratio (the ratio of phosphoprotein to total protein) of S342, but elevated that of S448, concomitant with the increase in total NEDD4-2 protein level, which were reversed by PLPP/CIN overexpression. Since NEDD4-2 phosphorylation inhibits ubiquitinations of various ion channel and maintains its protein level^12,21,23,25,32–34,39,40^, it is plausible that PLPP/CIN would inhibit NEDD4-2 E3 ubiquitin ligase activity by reducing target channel-NEDD4-2 interaction and its protein level by increasing S342 and diminishing S448 phosphorylation ratio, respectively. However, the present data could not provide the evidence for the role of S342 phosphorylation ratio in NEDD4-2-substrate interactions. Furthermore, the present study could not verify whether NEDD4-2 ubiquitin modification results from self-ubiquitination or the activations of other unknown E3 ligases, although NEDD4-2 is auto-ubiquitinated like other E3 ligases^32,33^. Thus, it would be needed to elucidate the role of the relative NEDD4-2 phosphorylation ratio in its activity, and the specific E3 ligase for NEDD4-2 ubiquitination in future studies.

NEDD4-2-mediated ubiquitination is one of the underlying machineries for synaptic homeostatic scaling, which is a feedback mechanism for maintenance of a physiological rage of neuronal excitability^24,42,43^. Thus, perturbation of the homeostatic scaling following stimulation may trigger a prolonged neuronal hyperexcitability inducing seizure activity^24^. In the present study, PLPP/CIN deletion interrupted seizure progression induced by KA, which was reversed by NEDD4-2 knockdown. Consistent with the present data, *Nedd4-2*^*andi*^ mice show the elevated KA-induced seizure susceptibility^13,14^. The present study also reveals that the enhancements of PLPP/CIN-NEDD4-2 bindings and NEDD4-2 ubiquitination in *PLPP/CIN*^*Tg*^ mice increased seizure intensity and its progression in response to KA. Thus, our findings indicate that PLPP/CIN-mediated NEDD4-2 ubiquitination may enhance seizure activity and lead to seizure progression.

Recently, it has been reported that the higher seizure susceptibility due to insufficient function of NEDD4-2 in *Nedd4-2*^*andi*^ mice is recovered by the genetically reducing GluA1 level^14^. Furthermore, *Fmr1*^*−/−*^ mice (the fragile X syndrome mouse model) show neuronal hyperexcitability, because loss-of-function dephosphorylation of NEDD4-2 impairs NEDD4-2-GluA1 interaction and GluA1 ubiquitination after chronic activity stimulation, but not under physiological condition^24^. Therefore, it is likely that NEDD4-2 may play an important role in downregulation of synaptic homeostatic scaling via GluA1 ubiquitination^13,14^. In the present study, PLPP/CIN regulated membrane GluA1 expression and its ubiquitination under physiological condition. Furthermore, PLPP/CIN increased the seizure intensity due to the reduced GluA1 ubiquitination following KA injection. Since PLPP/CIN enhances the synaptic strength during high-frequency stimulation^28^, our findings suggest a novel mechanism by which PLPP/CIN inhibits synaptic downscaling by dephosphorylating NEDD4-2 S448 site and disrupting the subsequent GluA1 ubiquitination during neuronal hyperexcitability. Conversely, it is plausible that GluA1-NEDD4-2 bindings would be affected by GluA1 phosphorylation level, since the Na_v_1.6 S553 phosphorylation by p38 mitogen-activated protein kinase is required for its binding to NEDD4-2^44^. Under physiological conditions, however, PLPP/CIN deletion/overexpression cannot affect GluA1 S831 and S845 phosphorylation levels^28^. Therefore, our findings also indicate that NEDD4-2-mediated GluA1 ubiquitination may be independent of GluA1 phosphorylation.

In the case of *Fmr1*^*−/−*^ mice^24^, the activity-dependent dephosphorylation of NEDD4-2 is caused by p53-medaited AKT destabilization, which is an upstream pathway of SGK1^12,45^. In the present study, KA reduced SGK1 activity and S342 and S448 phosphorylations of NEDD4-2. Thus, it is likely that activity-dependent SGK1 inhibition would also be involved in NEDD4-2 dephosphorylation. However, PLPP/CIN expression level influenced only NEDD4-2 S448 phosphorylation, but not NEDD4-2 S342 and SGK1 phosphorylation, under physiological and postseizure conditions. Therefore, our findings suggest that PLPP/CIN may fine-tune NEDD4-2-GluA1 interaction and GluA1 ubiquitination by S448 dephosphorylation under basal and pathological conditions independent of SGK1 activity.

Impaired KCNQ regulation causes spontaneous seizures due to reduction in M-current that exerts a stabilizing effect on the resting membrane potential and attenuates neuronal excitability^46,47^. NEDD4-2 phosphorylation at S448 site is essential for the SGK1-dependent regulation of KCNQ2/3 and KCNQ3/5 channels in *Xenopus* oocytes^39^. Considering E3 ubiquitin ligase activity of NEDD4-2 for KCNQ^34^, it is likely that PLPP/CIN-mediated NEDD4-2 ubiquitination would affect the surface KCNQ2/3/5 expressions. However, PLPP/CIN deletion/overexpression and NEDD4-2 knockdown did not show any significant effect on total or surface KCNQ2/3/5 expression, suggesting the dominant role of NEDD4-2 in GluA1 ubiquitination. These data demonstrate that PLPP/CIN may not influence the general ubiquitination process mediated by NEDD4-2. Since NEDD4-2 ubiquitinates multiple membrane receptors, including voltage-gated Na^+^ channel^16^, neurotrophin receptors^17^, and glutamate transporters^15^, however, the possibility is not excluded that changes in ubiquitination of other substrates could contribute to seizure activity when PLPP/CIN are deleted or overexpressed. Thus, further studies are needed to elucidate whether and how PLPP/CIN mediates ubiquitination status of other NEDD4-2 substrates, which contributes to seizure generation and/or its progression.

On the other hand, Zhu et al.^14^ have revealed that NEDD4-2 mutation increases seizure susceptibility in response to KA. Interestingly, the same group has also reported that murine double minute-2 triggers the degradation of p53 and subsequent induction of NEDD4-2 as an adaptive response in neuronal culture upon chronic elevation of neuronal activity by picrotoxin, a GABA_A_ receptor antagonist^13,38^. In the present study, however, NEDD4-2 expression level was reduced in the hippocampus of WT mice 2 h after KA injection. Similar to the present data, pilocarpine (a muscarinic acetylcholine receptor agonist)-induced acute seizure activity rapidly degrades NEDD4-2 in the rat hippocampus 2 h after injection^48^. These discrepancies would result from the differences in methods (neuronal culture study vs. in vivo study), seizure severity in response to convulsants (picrotoxin vs. pilocarpine or KA), or dose of KA (60 vs. 25 mg/kg). However, the data from these previous reports and the present study identically suggest that the reduced NEDD4-2 expression may increase seizure severity and its duration. Indeed, chronic epilepsy rat shows downregulation of NEDD4-2 expression^48^, and NEDD4-2 knockdown increased seizure severity and its duration in *PLPP/CIN*^*−/−*^ mice in the present study. Furthermore, the present data demonstrate that PLPP/CIN activity in *PLPP/CIN*^*Tg*^ mice was higher than that in WT mice under physiological condition. PLPP/CIN overexpression also diminished NEDD4-2 protein level with enhancing NEDD4-2 S448 dephosphorylation and its ubiquitination under physiological condition. In addition, KA enhanced NEDD4-2 S448 dephosphorylation in WT and *PLPP/CIN*^*Tg*^ mice without altering PLPP/CIN activity, indicating that seizure activity may increase NEDD4-2 dephosphorylation via promoting physical interaction between PLPP/CIN and NEDD4-2. Therefore, our findings suggest that PLPP/CIN-mediated NEDD4-2 dephosphorylation may play an important role in the seizure intensity and its progression, regardless of the presence of compensatory upregulation of NEDD4-2 expression in response to seizures^13,38,48^.

In conclusion, we describe a new paradigm for the regulation of NEDD4-2 activity by PLPP/CIN-mediated S448 dephosphorylation (Fig. 8c). To our knowledge, these findings provide the novel PLPP/CIN-mediated mechanism underlying seizure progression by modulating NEDD4-2-mediated GluA1 ubiquitination and suggest the development of therapeutic strategies for various neurological and psychiatric disorders, including epilepsy.

## Methods

### Experimental animals and chemicals

*PLPP/CIN*^*−/−*^ (129/SvEv-C57BL/6J background) and *PLPP/CIN*^*Tg*^ (C57BL/6J background) mice were used in the present study. Each background WT mice were used as control animals, respectively. Animals were provided with a commercial diet and water ad libitum under controlled temperature, humidity, and lighting conditions (22 ± 2 °C, 55 ± 5%, and a 12:12 light/dark cycle). All experimental protocols were approved by the Animal Care and Use Committee of Hallym University. All reagents were obtained from Sigma-Aldrich (St. Louis, MO, USA), except as noted.

### In vitro PLPP/CIN phosphatase assay

Modified in vitro PLPP/CIN phosphatase assay using full-length recombinant human NEDD4-2 (Abcam, UK) and human PLPP/CIN proteins (Abcam, UK) was performed as described previously^30^. NEDD4-2 (10 ng/μl) was phosphorylated by incubation with 200 U/μl SGK1 (SignalChem, Canada) and 100 μM ATP in the kinase assay buffer I (SignalChem, Canada) at 30 °C for 1 h. Thereafter, the sample was portioned the same volume, added PLPP/CIN (10 ng/μl) or 50 mM Tris buffer (control), and incubated at 30 °C for 1 h. Crude extracts obtained from the same *PLPP/CIN*^*−/−*^ mice were used the same method omitting SGK1 and NEDD4-2. Thereafter, the samples were used coprecipitation and western blot analysis (see below).

### NEDD4-2 knockdown and electrode implantation

Under anesthesia with isoflurane (3% induction, 1.5–2% for surgery and 1.5% maintenance in a 65:35 mixture of N_2_O:O_2_). Surgery for a brain infusion kit and an electrode implantation was performed according to our previous study^4,30^. A brain kit 3 (Alzet, USA) was inserted into the lateral cerebral ventricle (2.0 mm depth from bregma), and connected with a 1007D Alzet osmotic pump (Alzet, USA) containing control siRNA (20 μM) or NEDD4-2 siRNA (20 μM), respectively. siRNA sequence targeting NEDD4-2 corresponding to coding region (5′→3′) are sense: AAACUCUCUGGAGUACGGAACAGCCUU, and antisense: GGCUGUUCCGUACUCCAGAGAGUUUUU, or sense: UUCAGAUCCACUUGGUAUGUCUGCCUU, and antisense: GGCAGACAUACCAAGUGGAUCUGAAUU. Non-silencing RNA (5′-GGCGCGCTTTGTAGGATTCGA-3′) was used as the control siRNA (Genolution, South Korea). Osmotic pump was implanted subcutaneously in the midscapular region of the back. Monopolar electrode (Plastics One, USA) or a guide-electrode-combo (C313G-MS303/2/SPC, Plastics One, USA) was also implanted into the left dorsal hippocampus (2 mm posterior; 1.25 mm lateral; 2 mm depth from bregma).

### Seizure induction and EEG Recording

After baseline recording for at least 30 min, animals were given KA (25 mg/kg, i.p.). Control animals received an equal volume of normal saline instead of KA. EEG signals were recorded with a DAM 80 differential amplifier (0.1–1000 Hz bandpass; World Precision Instruments, USA) and the data were digitized (1000 Hz) and analyzed using LabChart Pro v7 software (AD Instruments, Australia). Latency of seizure onset was defined as the time point showing more than 3 s and consisting of a rhythmic discharge between 4 and 10 Hz with amplitude of at least two times higher than the baseline EEG^4,30^. Total EEG power was normalized by the baseline power obtained from each animal. Spectrograms were automatically calculated using a Hanning sliding window with 50% overlap by LabChart Pro v7. Diazepam (Valium; Roche, France; 10 mg/kg, i.p.) was administered 2 h after KA injection and repeated, as needed. After recording, animals were quickly decapitated, and their hippocampi were dissected out in the presence of cooled artificial cerebrospinal fluid (in mM: 124 NaCl, 5 KCl, 1.25 NaH_2_PO_4_, 26 NaHCO_3_, 10 dextrose, 1.5 MgCl_2_, and 2.5 CaCl_2_) and stored −80 °C until preparation for biochemical experiments^30^.

### Analysis of neuronal activity in responses to AMPA

After baseline recording for at least 30 min, animals implanted with a guide-electrode-combo (C313G-MS303/2/SPC, Plastics One, USA) were directly infused AMPA (20 μM) over a 1-min period using a microinjection pump (1 μl/min, KD Scientific, USA) into the hippocampus with an internal infusion cannula (C315IA, Plastics One, USA)^29^. EEG signals were digitized and analyzed using LabChart Pro v7 (AD Instruments) by the same methods aforementioned.

### Co-immunoprecipitation and membrane fraction

The hippocampal tissues were lysed in the radioimmunoprecipitation assay buffer (RIPA: 50 mM Tris–HCl pH 8.0; 1% Nonidet P-40; 0.5% deoxycholate; 0.1% SDS, Thermo Fisher Scientific, USA) containing protease inhibitor cocktail (Roche Applied Sciences, USA), phosphatase inhibitor cocktail (PhosSTOP^®^, Roche Applied Science, USA) and 1 mM sodium orthovanadate. Protein concentrations were calibrated by the BCA protein assay (Pierce, USA) and equal amounts of total proteins were incubated with NEDD4-2, Ubiquitin, or GluA1 antibody (Supplementary Table 1) and protein G sepharose beads at 4 °C overnight. Beads were collected by centrifugation, eluted in 2 × SDS sample buffer, and boiled at 95 °C for 5 min. To analyze membrane expressions of KCNQ and GluA1, we used a subcellular Protein Fractionation Kit for Tissues (Thermo Scientific, USA), according to the manufacturer’s instructions.

### PLPP/CIN activity assay

The hippocampal tissues were homogenized in TE buffer. Total protein content was measured by the BCA protein assay kit. To measure PLPP/CIN activity in lysates, we used PLPP/CIN activity assay kits (#OKEH01229 and #OKEH03398; Aviva systems biology, USA), according to the manufacturer’s instructions.

### Western blot

Western blotting was performed according to standard procedures. The list of primary antibody used in the present study is Supplementary Table 1. The rabbit anti-β-actin (input) or N-cadherin (membrane fraction) was used as internal reference. The signals were scanned and quantified on ImageQuant LAS 4000 system (GE health, USA). The values of each sample were normalized with the corresponding amount of β-actin or N-cadherin.

### Fluoro-Jade B (FJB) Staining and cell counting

One day after KA injection, animals were perfused transcardially with 4% paraformaldehyde in 0.1 M phosphate buffer (pH 7.4) under urethane anesthesia (1.5 g/kg, i.p.). Brains were postfixed in the same fixative overnight and then cryoprotected and sectioned at 30 μm with a cryostat. Thereafter, tissues were used for a conventional FJB staining according to previous studies^49,50^. All images were obtained using an AxioImage M2 microscope and AxioVision Rel. 4.8 software. Areas of interest (1 × 10^5^ μm^2^) were selected in the captured images of the CA3 region of the hippocampus proper (ten sections per each animal), and the number of FJB-positive neurons was counted^49,50^.

### Statistical analysis

After evaluating the values on normality using Shapiro–Wilk *W*-test, Student’s *t* test or ANOVA were used to analyze statistical significance. Bonferroni’s test was applied for *post hoc* comparisons. A *p*-value below 0.05 was considered statistically significant. Quantitative data were expressed as mean ± standard error of the mean.

## Supplementary information

Supplemental material

## References

[1] Elger CE, Helmstaedter C, Kurthen M (2004). Chronic epilepsy and cognition. Lancet Neurol..

[2] Seeburg DP, Sheng M (2008). Activity-induced Polo-like kinase 2 is required for homeostatic plasticity of hippocampal neurons during epileptiform activity. J. Neurosci..

[3] Mantegazza M, Curia G, Biagini G, Ragsdale DS, Avoli M (2010). Voltage-gated sodium channels as therapeutic targets in epilepsy and other neurological disorders. Lancet Neurol..

[4] Kim JE, Kang TC (2011). The P2X7 receptor-pannexin-1 complex decreases muscarinic acetylcholine receptor-mediated seizure susceptibility in mice. J. Clin. Investig..

[5] Kim JE (2008). Potential role of pyridoxal-5′-phosphate phosphatase/chronophin in epilepsy. Exp. Neurol..

[6] Kang TC (2006). Epileptogenic roles of astroglial death and regeneration in the dentate gyrus of experimental temporal lobe epilepsy. Glia.

[7] McNamara JO, Huang YZ, Leonard AS (2006). Molecular signaling mechanisms underlying epileptogenesis. Sci. STKE.

[8] Wetherington J, Serrano G, Dingledine R (2008). Astrocytes in the epileptic brain. Neuron.

[9] Vezzani A, French J, Bartfai T, Baram TZ (2011). The role of inflammation in epilepsy. Nat. Rev. Neurol..

[10] Hallengren J, Chen PC, Wilson SM (2013). Neuronal ubiquitin homeostasis. Cell Biochem. Biophys..

[11] Schwarz LA, Patrick GN (2012). Ubiquitin-dependent endocytosis, trafficking and turnover of neuronal membrane proteins. Mol. Cell. Neurosci..

[12] Chandran S (2011). Neural precursor cell-expressed developmentally down-regulated protein 4-2 (Nedd4-2) regulation by 14-3-3 protein binding at canonical serum and glucocorticoid kinase 1 (SGK1) phosphorylation sites. J. Biol. Chem..

[13] Jewett KA (2016). Feedback modulation of neural network synchrony and seizure susceptibility by Mdm2-p53-Nedd4-2 signaling. Mol. Brain.

[14] Zhu J (2017). Epilepsy-associated gene Nedd4-2 mediates neuronal activity and seizure susceptibility through AMPA receptors. PLoS Genet.

[15] Zhang Y (2017). Regulation of glutamate transporter trafficking by Nedd4-2 in a Parkinson’s disease model. Cell Death Dis..

[16] Ekberg JA (2014). Nedd4-2 (NEDD4L) controls intracellular Na(+)-mediated activity of voltage-gated sodium channels in primary cortical neurons. Biochem. J..

[17] Georgieva MV, de Pablo Y, Sanchis D, Comella JX, Llovera M (2011). Ubiquitination of TrkA by Nedd4-2 regulates receptor lysosomal targeting and mediates receptor signaling. J. Neurochem..

[18] Allen AS (2013). De novo mutations in epileptic encephalopathies. Nature.

[19] Dibbens LM (2007). NEDD4-2 as a potential candidate susceptibility gene for epileptic photosensitivity. Genes Brain Behav..

[20] Vanli-Yavuz EN (2015). Investigation of the possible association of NEDD4-2 (NEDD4L) gene with idiopathic photosensitive epilepsy. Acta Neurol. Belg..

[21] Ichimura T (2005). 14-3-3 proteins modulate the expression of epithelial Na^+^ channels by phosphorylation-dependent interaction with Nedd4-2 ubiquitin ligase. J. Biol. Chem..

[22] Bhalla V (2005). Serum- and glucocorticoid-regulated kinase 1 regulates ubiquitin ligase neural precursor cell-expressed, developmentally down-regulated protein 4-2 by inducing interaction with 14-3-3. Mol. Endocrinol..

[23] Bhalla V (2006). AMP-activated kinase inhibits the epithelial Na^+^ channel through functional regulation of the ubiquitin ligase Nedd4-2. J. Biol. Chem..

[24] Lee KY, Jewett KA, Chung HJ, Tsai NP (2018). Loss of fragile X protein FMRP impairs homeostatic synaptic downscaling through tumor suppressor p53 and ubiquitin E3 ligase Nedd4-2. Hum. Mol. Genet.

[25] Ho PY (2018). β(1)Pix exchange factor stabilizes the ubiquitin ligase Nedd4-2 and plays a critical role in ENaC regulation by AMPK in kidney epithelial cells. J. Biol. Chem..

[26] Debonneville C (2001). Phosphorylation of Nedd4-2 by Sgk1 regulates epithelial Na^+^ channel cell surface expression. EMBO J..

[27] Lee IH, Dinudom A, Sanchez-Perez A, Kumar S, Cook DI (2007). Akt mediates the effect of insulin on epithelial sodium channels by inhibiting Nedd4-2. J. Biol. Chem..

[28] Kim JE (2016). PLPP/CIN regulates bidirectional synaptic plasticity via GluN2A interaction with postsynaptic proteins. Sci. Rep..

[29] Jeon AR, Kim JE (2018). PDI knockdown inhibits seizure activity in acute seizure and chronic epilepsy rat models via S-nitrosylation-independent thiolation on NMDA receptor. Front. Cell. Neurosci..

[30] Kim JE (2017). PLPP/CIN regulates seizure activity by the differential modulation of calsenilin binding to GluN1 and Kv4.2 in mice. Front. Mol. Neurosci..

[31] Hayashi M (2001). BMK1 mediates growth factor-induced cell proliferation through direct cellular activation of serum and glucocorticoid-inducible kinase. J. Biol. Chem..

[32] Bruce MC (2008). Regulation of Nedd4-2 self-ubiquitination and stability by a PY motif located within its HECT-domain. Biochem J..

[33] Cui Z, Zhang S (2013). Regulation of the human ether-a-go-go-related gene (hERG) channel by Rab4 protein through neural precursor cell-expressed developmentally down-regulated protein 4-2 (Nedd4-2). J. Biol. Chem..

[34] Ekberg J (2007). Regulation of the voltage-gated K^+^ channels KCNQ2/3 and KCNQ3/5 by ubiquitination. Novel role for Nedd4-2. J. Biol. Chem..

[35] Zara F (2013). Genetic testing in benign familial epilepsies of the first year of life: clinical and diagnostic significance. Epilepsia.

[36] Allen NM (2014). The variable phenotypes of KCNQ-related epilepsy. Epilepsia.

[37] Soldovieri MV (2016). Early-onset epileptic encephalopathy caused by a reduced sensitivity of Kv7.2 potassium channels to phosphatidylinositol 4,5-bisphosphate. Sci. Rep..

[38] Jewett KA, Zhu J, Tsai NP (2015). The tumor suppressor p53 guides glua1 homeostasis through Nedd4-2 during chrnoic elevation of neuronal activity. J. Neurochem..

[39] Schuetz F, Kumar S, Poronnik P, Adams DJ (2008). Regulation of the voltage-gated K^+^ channels KCNQ2/3 and KCNQ3/5 by serum- and glucocorticoid-regulated kinase-1. Am. J. Physiol. Cell Physiol..

[40] Abriel H, Kamynina E, Horisberger JD, Staub O (2000). Regulation of the cardiac voltage‐gated Na^+^ channel (H1) by the ubiquitin‐protein ligase Nedd4. FEBS Lett..

[41] Boehmer C (2006). Post-translational regulation of EAAT2 function by co-expressed ubiquitin ligase Nedd4-2 is impacted by SGK kinases. J. Neurochem.

[42] Turrigiano G (2012). Homeostatic synaptic plasticity: local and global mechanisms for stabilizing neuronal function. Cold Spring Harb. Perspect. Biol..

[43] Davis GW (2013). Homeostatic signaling and the stabilization of neural function. Neuron.

[44] Gasser A (2010). Two Nedd4-binding motifs underlie modulation of sodium channel Nav1.6 by p38 MAPK. J. Biol. Chem..

[45] Singh PK, Singh S, Ganesh S (2013). Activation of serum/glucocorticoid-induced kinase 1 (SGK1) underlies increased glycogen levels, mTOR activation, and autophagy defects in Lafora disease. Mol. Biol. Cell.

[46] Maslarova A (2013). Increased susceptibility to acetylcholine in the entorhinal cortex of pilocarpine-treated rats involves alterations in KCNQ channels. Neurobiol. Dis..

[47] Brown DA, Adams PR (1980). Muscarinic suppression of a novel voltage-sensitive K^+^ current in a vertebrate neurone. Nature.

[48] Wu L (2015). The role of ubiquitin/Nedd4-2 in the pathogenesis of mesial temporal lobe epilepsy. Physiol. Behav..

[49] Kim JE, Kang TC (2018). Differential roles of mitochondrial translocation of active caspase-3 and HMGB1 in neuronal death induced by status epilepticus. Front. Cell. Neurosci..

[50] Ko AR, Kang TC (2017). TRPC6-mediated ERK1/2 phosphorylation prevents dentate granule cell degeneration via inhibiting mitochondrial elongation. Neuropharmacology.

