# Supplementary material for: PLPP/CIN-mediated NEDD4-2 S448 dephosphorylation regulates neuronal excitability via GluA1 ubiquitination
Source: Cell Death Dis. 2019 Jul 18;10(8):545. doi: 10.1038/s41419-019-1781-0 (PMC6639327; doi:10.1038/s41419-019-1781-0)
Supplement: Supplementary file 1 — Supplemental material [file 41419_2019_1781_MOESM1_ESM.docx]

**Supporting information**

**PLPP/CIN-mediated NEDD4-2 S448 dephosphorylation regulates neuronal excitability via GluA1 ubiquitination**

Ji-Eun Kim,^1^ Duk-Shin Lee, ^1^ A Ran Jeon, ^1^ Min Ju Kim, ^1^ Tae-Cheon Kang^1,*^

^1^Department of Anatomy and Neurobiology, Institute of Epilepsy Research, College of Medicine, Hallym University, Chunchon 24252, South Korea

* Correspondence to: T. -C. Kang, Department of Anatomy and Neurobiology, College of Medicine, Hallym University, Chunchon, Kangwon-Do 24252, South Korea; Tel: +82-33-248-2524; Fax: +82-33-248-2525; E-mail: tckang@hallym.ac.kr

**Supplementary Table 1. Primary antibodies used in the present study**

| Antigen | Host | Manufacturer  (catalog number) | Dilution used |
| --- | --- | --- | --- |
| GluA1 | Mouse | Synaptic systems (182011) | 1:100 (IP)  1:1000 (WB) |
| KCNQ2 | Rabbit | Alomone labs (APC-050)  Cell signaling technology  (#14752) | 1:400 (WB)  1:1000 (WB) |
| KCNQ3 | Rabbit | Alomone labs (APC-050) | 1:400 (WB) |
| KCNQ5 | Rabbit | Alomone labs (APC-050) | 1:400 (WB) |
| N-cadherin | Rabbit | Abcam (ab18203) | 1:4000 (WB) |
| NEDD4-2 | Rabbit | Abcam (ab131167): IP  Abcam (ab46521): WB | 1:100 (IP)  1:1000 (WB) |
| PLPP/CIN | Rabbit | Sigma (HPA001099) | 1:1,000 (WB) |
| pNEDD4-2 (mouse S328, equivalent to human S448) | Rabbit | Abcam (ab95399) | 1:1,000 (WB) |
| pNEDD4-2 (S342) | Rabbit | Cell signaling technology  (#12146) | 1:1000 (WB) |
| pNEDD4-2 (S448) | Rabbit | Abcam (ab168349) | 1:1000 (WB) |
| pSGK1 (S422) | Rabbit | Abcam (ab55281) | 1:1000 (WB) |
| pSGK1 (S78) | Rabbit | Thermo (PA5-38392) | 1:1000 (WB) |
| SGK1 | Rabbit | ST John’s Laboratory  (STJ25513) | 1:100 (IP)  1:1000 (WB) |
| Ubiquitin | Rabbit | Genetex (GTX14372) |  |
| β-actin | Mouse | Sigma (A5316) | 1:5000 (WB) |

IP, Immunoprecipitation; WB, Western blot.


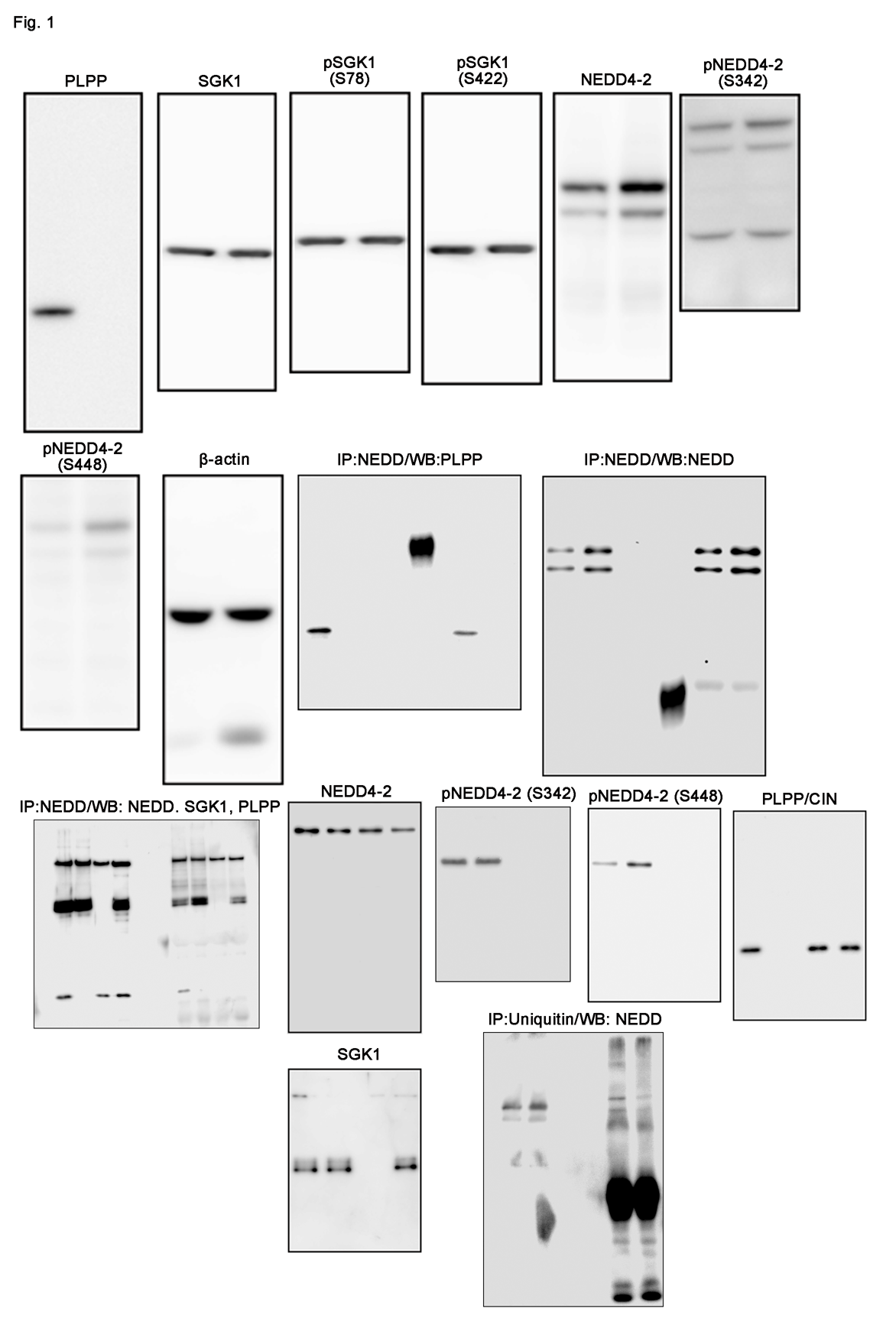


Supplementary Fig. 1. Full-length gel images of western blot data in Fig. 1.


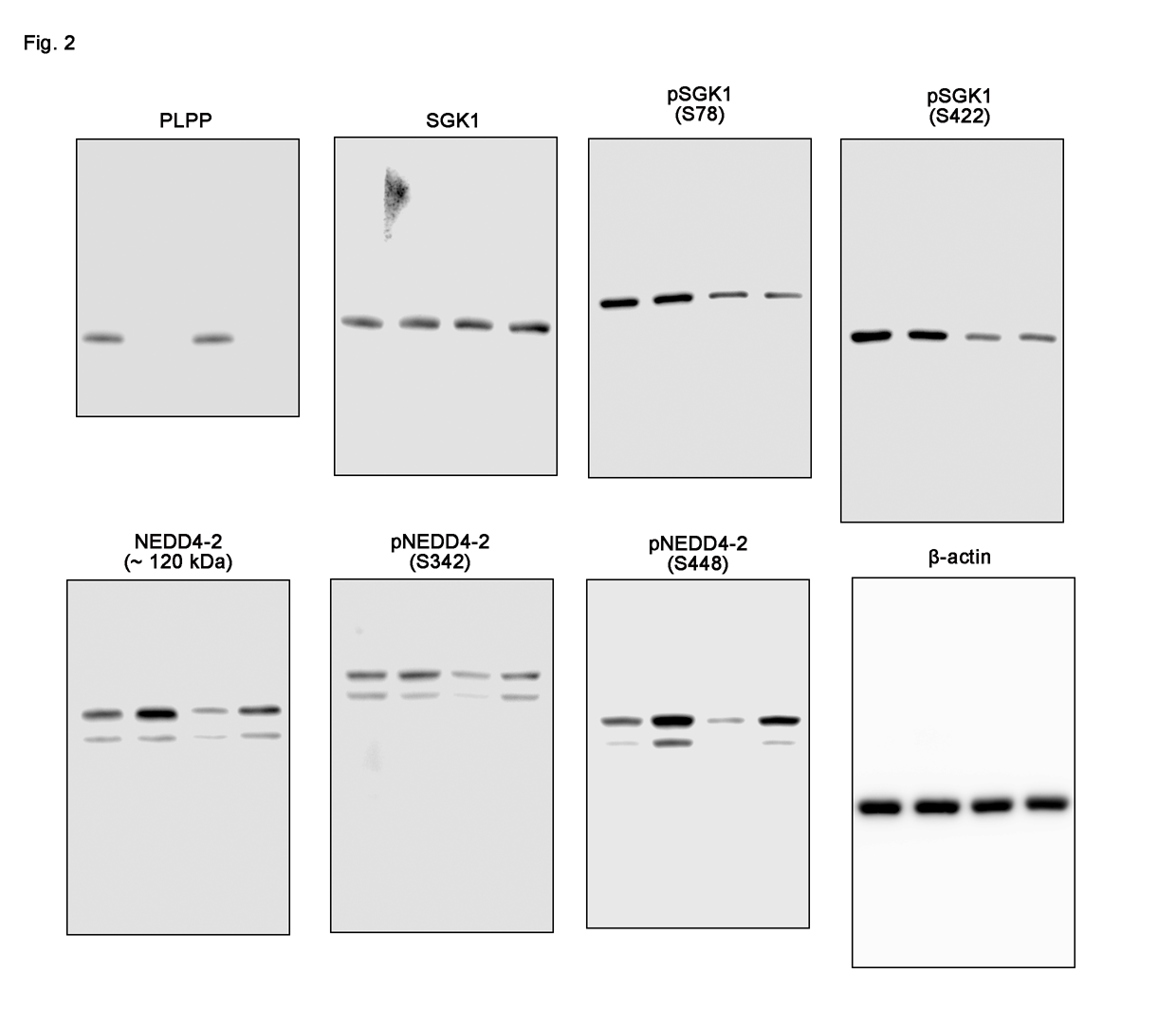


Supplementary Fig. 2. Full-length gel images of western blot data in Fig. 2.


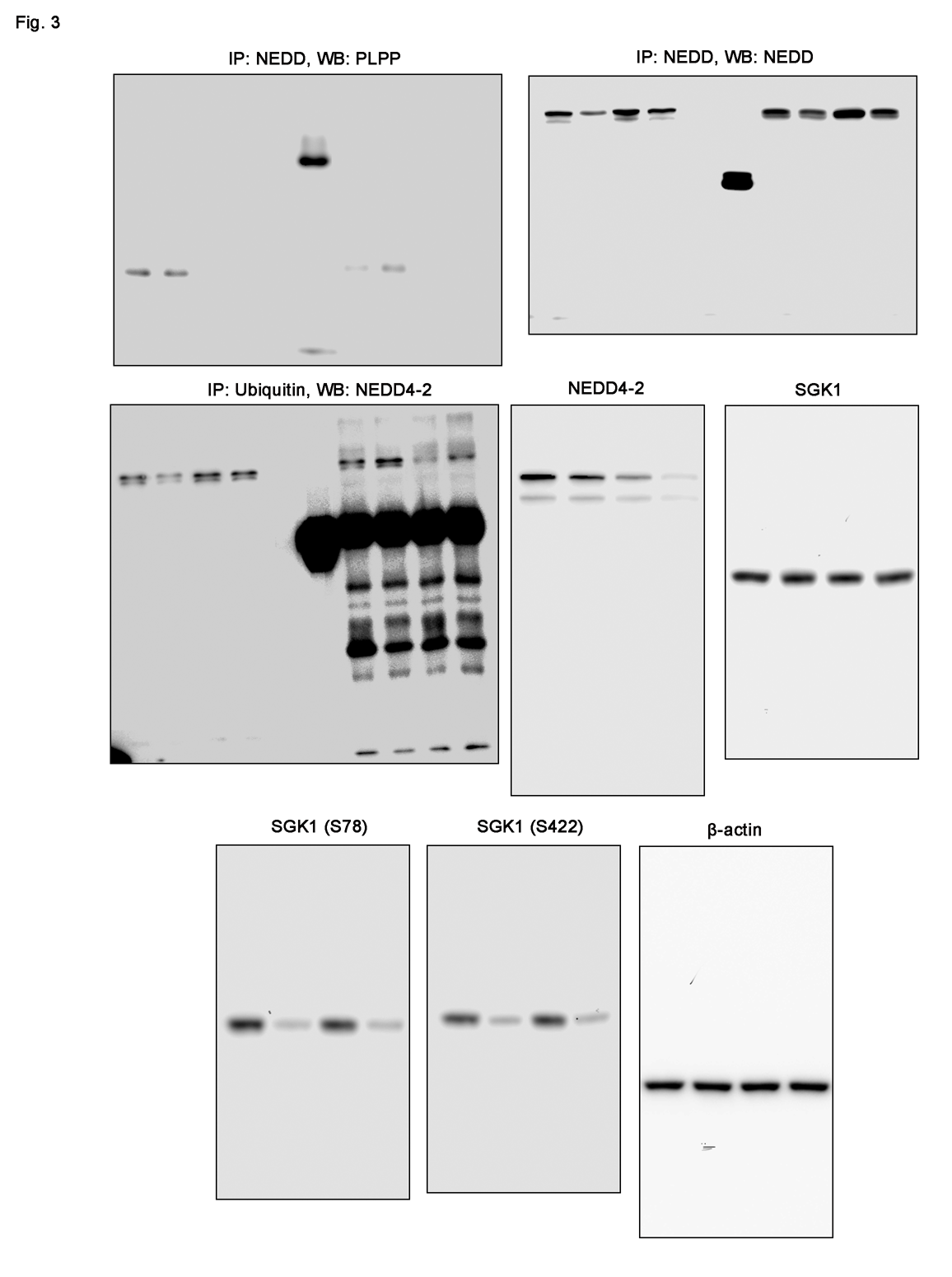


Supplementary Fig. 3. Full-length gel images of western blot data in Fig. 3.


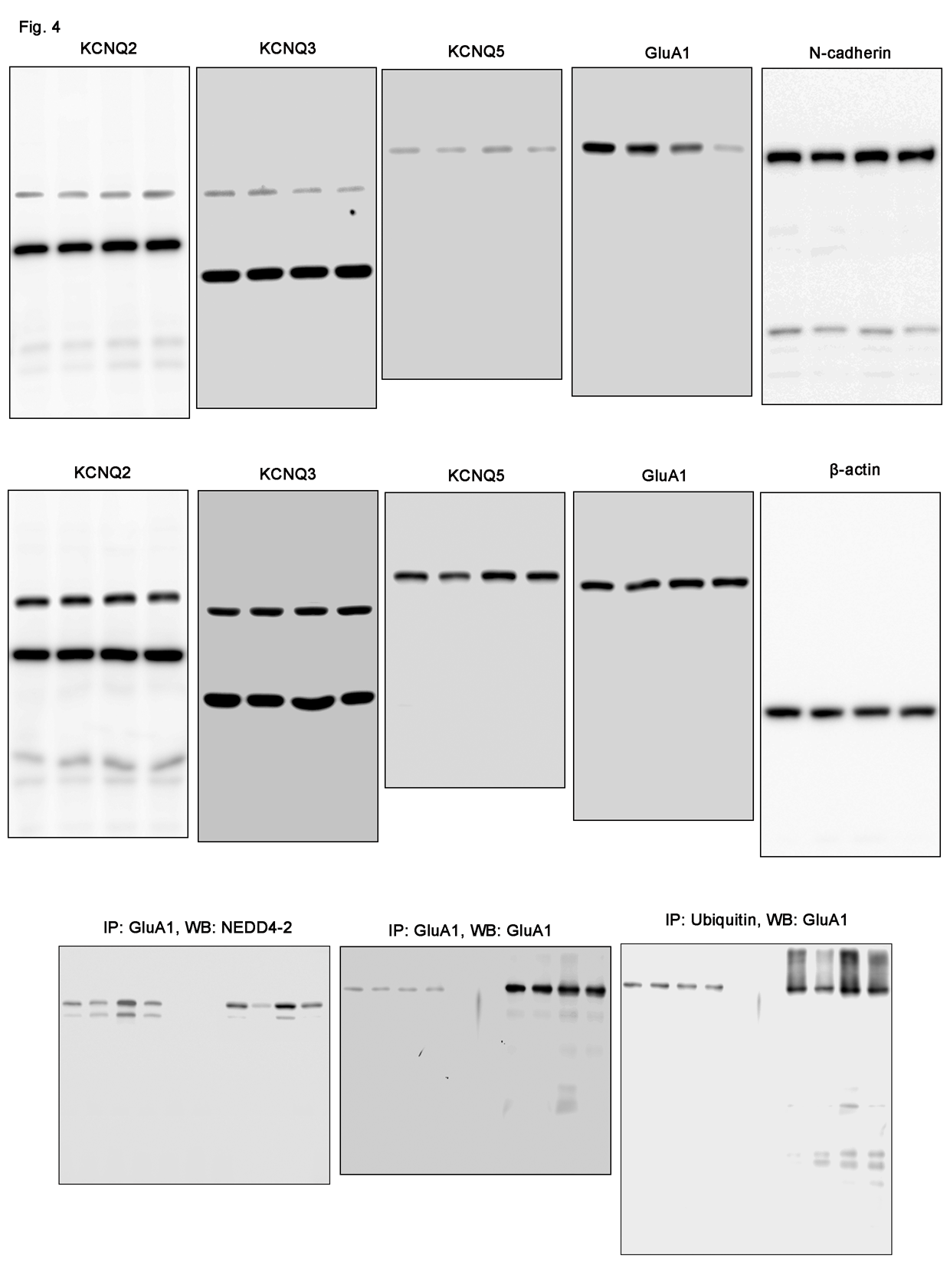


Supplementary Fig. 4. Full-length gel images of western blot data in Fig. 4.


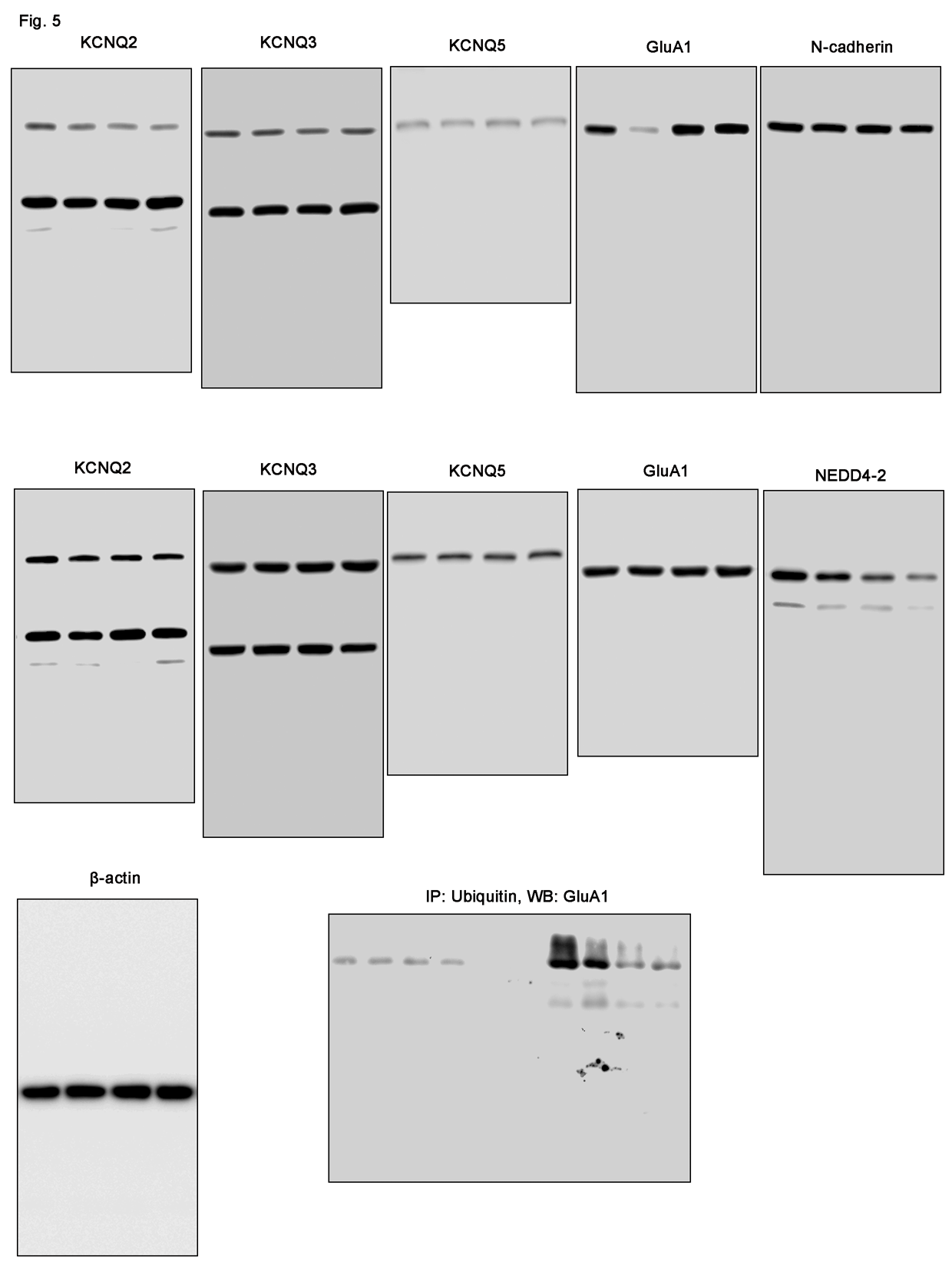


Supplementary Fig. 5. Full-length gel images of western blot data in Fig. 5.


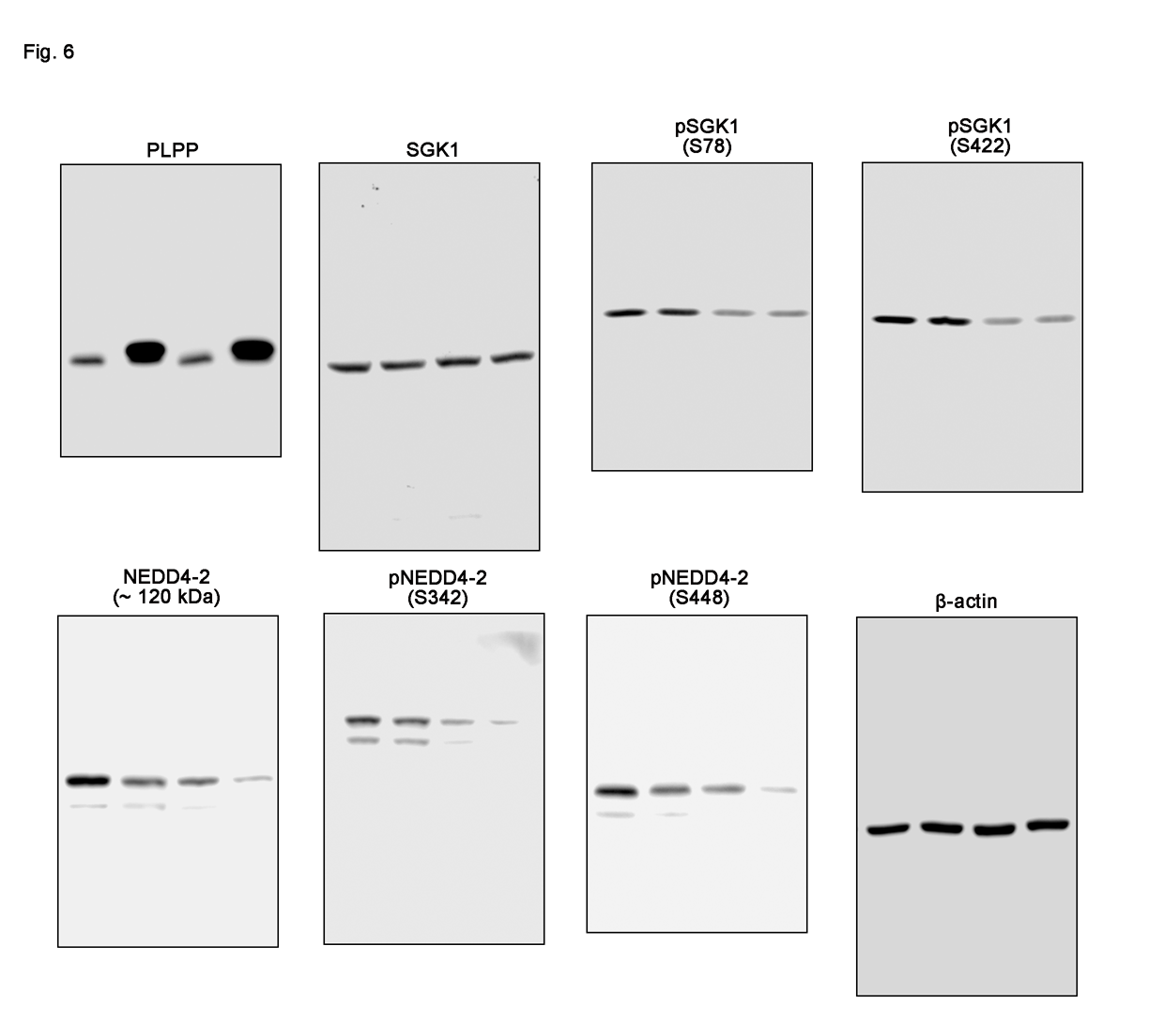


Supplementary Fig. 6. Full-length gel images of western blot data in Fig. 6.


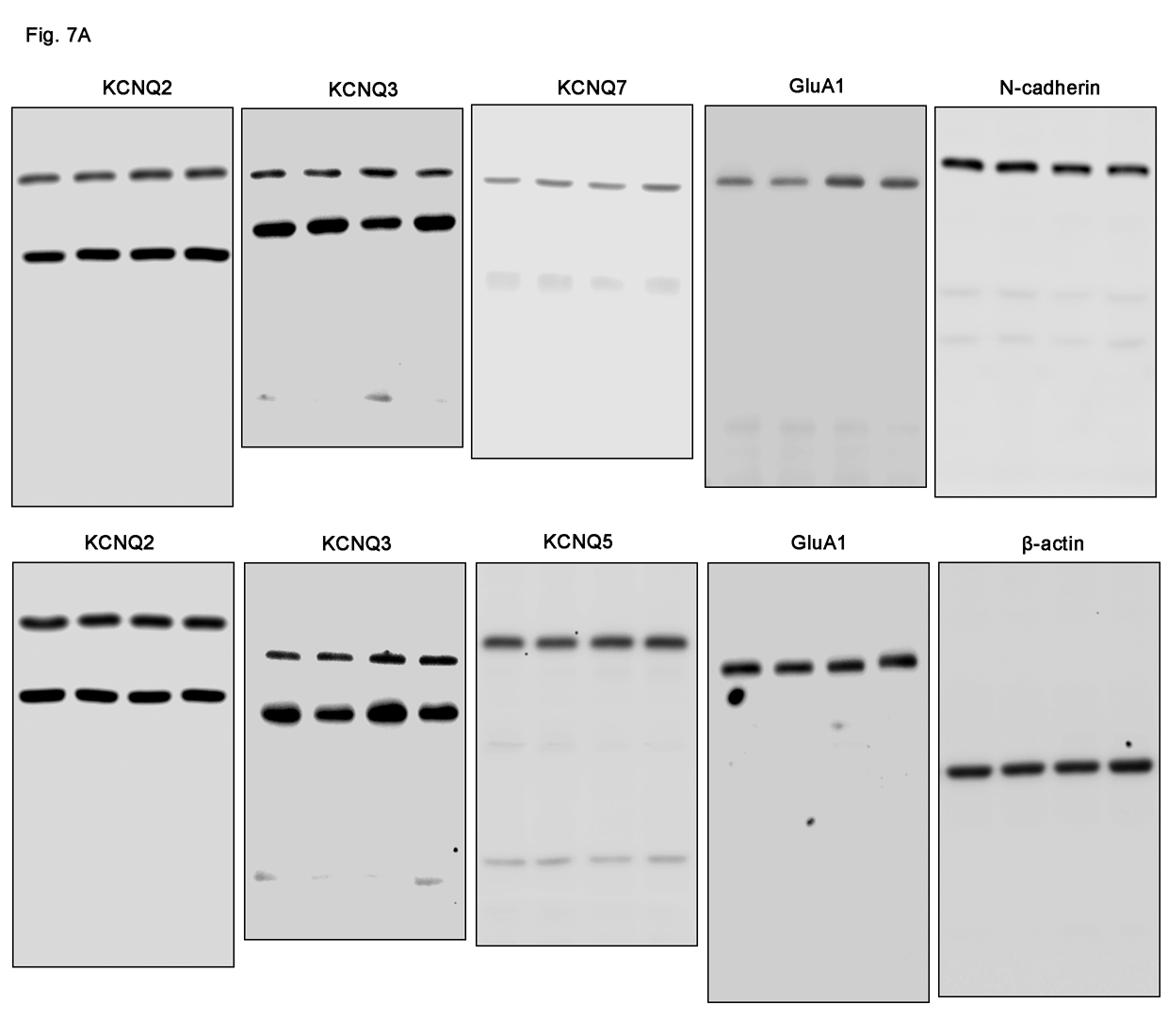


Supplementary Fig. 7. Full-length gel images of western blot data in Fig. 7A.


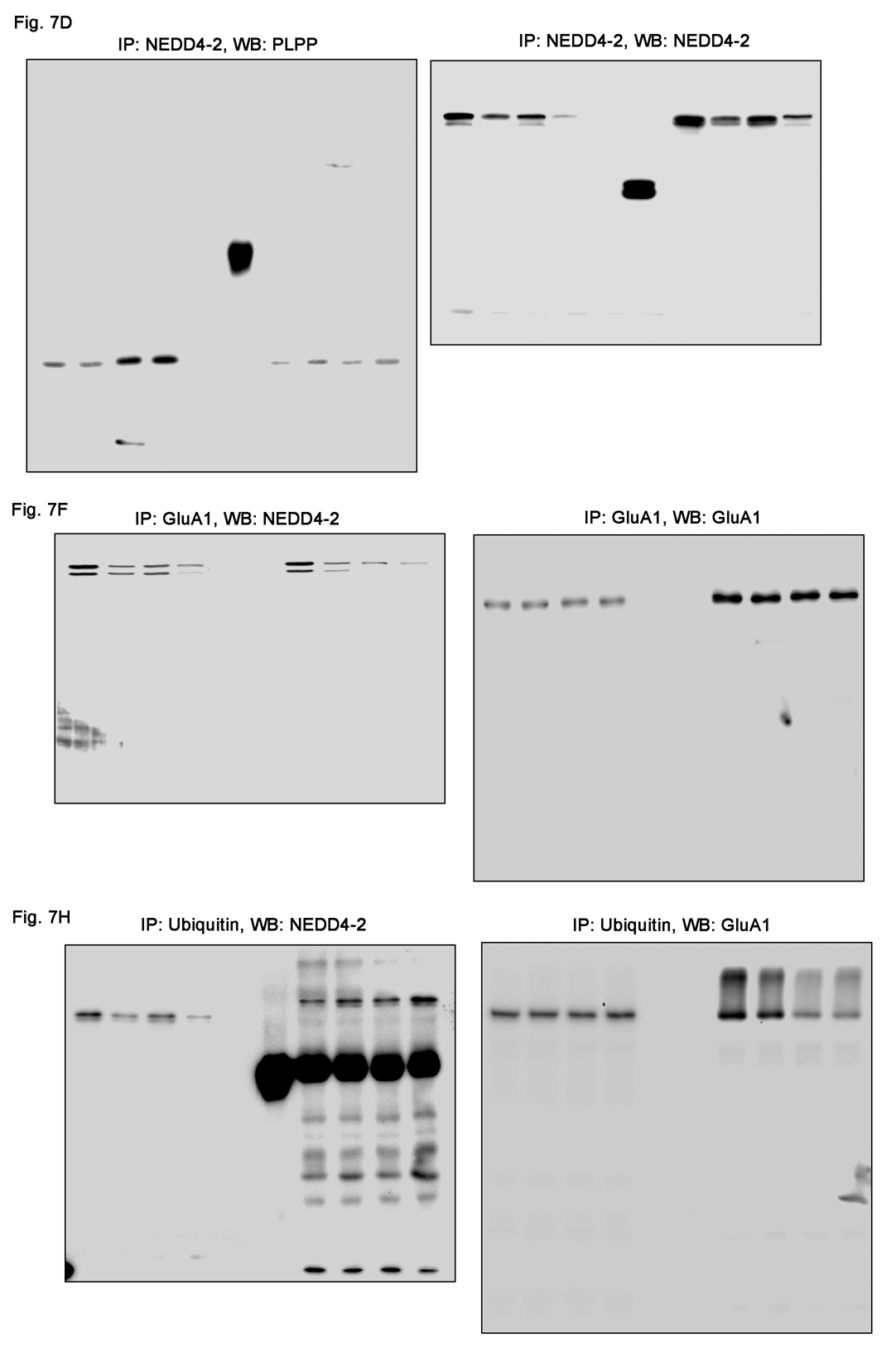


Supplementary Fig. 8. Full-length gel images of western blot data in Fig. 7D, 7F and 7H.
